# The Role of Uptake and Efflux Transporters in the Disposition of Glucuronide and Sulfate Conjugates

**DOI:** 10.3389/fphar.2021.802539

**Published:** 2022-01-13

**Authors:** Erkka Järvinen, Feng Deng, Wilma Kiander, Alli Sinokki, Heidi Kidron, Noora Sjöstedt

**Affiliations:** ^1^ Clinical Pharmacology, Pharmacy, and Environmental Medicine, Department of Public Health, University of Southern Denmark, Odense, Denmark; ^2^ Department of Clinical Pharmacology, Faculty of Medicine, University of Helsinki, Helsinki, Finland; ^3^ Individualized Drug Therapy Research Program, Faculty of Medicine, University of Helsinki, Helsinki, Finland; ^4^ Division of Pharmaceutical Biosciences, Faculty of Pharmacy, University of Helsinki, Helsinki, Finland

**Keywords:** ABC transporter, acyl glucuronide, drug-drug interaction (DDI), enterohepatic recycling, solute carrier, sulfotransferase (SULT), transporter inhibition, UDP-glucuronosyltransferase (UGT)

## Abstract

Glucuronidation and sulfation are the most typical phase II metabolic reactions of drugs. The resulting glucuronide and sulfate conjugates are generally considered inactive and safe. They may, however, be the most prominent drug-related material in the circulation and excreta of humans. The glucuronide and sulfate metabolites of drugs typically have limited cell membrane permeability and subsequently, their distribution and excretion from the human body requires transport proteins. Uptake transporters, such as organic anion transporters (OATs and OATPs), mediate the uptake of conjugates into the liver and kidney, while efflux transporters, such as multidrug resistance proteins (MRPs) and breast cancer resistance protein (BCRP), mediate expulsion of conjugates into bile, urine and the intestinal lumen. Understanding the active transport of conjugated drug metabolites is important for predicting the fate of a drug in the body and its safety and efficacy. The aim of this review is to compile the understanding of transporter-mediated disposition of phase II conjugates. We review the literature on hepatic, intestinal and renal uptake transporters participating in the transport of glucuronide and sulfate metabolites of drugs, other xenobiotics and endobiotics. In addition, we provide an update on the involvement of efflux transporters in the disposition of glucuronide and sulfate metabolites. Finally, we discuss the interplay between uptake and efflux transport in the intestine, liver and kidneys as well as the role of transporters in glucuronide and sulfate conjugate toxicity, drug interactions, pharmacogenetics and species differences.

## 1 Introduction

Metabolic enzymes and membrane transporters that are expressed in the intestine, liver and kidney have a significant impact on the absorption, distribution, metabolism and excretion of drugs and other compounds. Drug metabolism is mediated primarily by cytochrome P450 (CYP) enzymes (phase I metabolism) and conjugation reactions (phase II metabolism) catalyzed by uridine 5′-diphospho-glucuronosyltransferases (UGTs) and sulfotransferases (SULTs). UGT-mediated glucuronidation is a major metabolic pathway for 12% of drugs, while SULTs contribute major metabolites for ≈1% of drugs (Cerny, 2016). Furthermore, UGTs contribute to some extent to the metabolism of >50% of the 200 most prescribed drugs (Guillemette et al., 2014), evidencing the potential significance of UGTs on drug clearance and pharmacokinetics. Conjugation reactions are also important in steroid homeostasis in humans (Rižner, 2013) and in the elimination of natural compounds (e.g. flavonoids) consumed in food or as dietary supplements (Manach et al., 2004).

UGT- and SULT-mediated conjugation reactions introduce a negative charge and reduce lipid partitioning of the substrate by 2-5 logP units (Smith et al., 1985; Manners et al., 1988; Smith and Dalvie, 2012), which typically results in negligible passive permeability of the formed metabolite. This is in contrast to most phase I metabolites that possess higher lipophilicity than conjugates (Loi et al., 2013), and therefore the disposition of glucuronide and sulfate metabolites depends on organic anion transporters. Conjugated drug metabolites are considered to have a small impact on drug therapy, because they are typically pharmacologically inactive and facilitate drug excretion from the body. However, some phase II conjugates, such as reactive acyl glucuronide metabolites or glucuronides capable of enzyme inhibition, are known to affect drug efficacy and safety (Ogilvie et al., 2006; Regan et al., 2010; Tornio et al., 2014). Human esterases are capable of cleaving acyl glucuronides and releasing the parent compound from the conjugate (Fukami and Yokoi, 2012). Furthermore, the glucuronide or sulfate groups of drug conjugates can be cleaved by human β-glucuronidases or different sulfatases expressed in tissues, resulting in a release of the parent drug in tissues (Pang et al., 1994; Sperker et al., 1997; Mueller et al., 2015). For example, flavonoid glucuronides may be deconjugated in tissues and subsequently increase local parent exposure (Perez-Vizcaino et al., 2012). The highest deglucuronidation and desulfation activity in the body is found in the intestine within bacteria that express numerous different β-glucuronidases and sulfatases (Pollet et al., 2017; Ervin et al., 2020). Intestinal deconjugation may therefore also prolong parent drug exposure via enterohepatic recycling (Roberts et al., 2002). Thus, understanding the combined effects of uptake and efflux transport on phase II conjugates in different organs is important for predicting drug disposition and possible changes related to altered transporter function.

The aim of this review is to summarize knowledge on the uptake and efflux transport of glucuronide and sulfate conjugates. These conjugates are abbreviated as -G for glucuronide, -AG for acyl glucuronide and -S for sulfate. We focus on drug conjugates, but also include endogenous compounds and natural products. The review highlights the interplay between uptake and efflux in the liver and kidneys, and the role of transporters in glucuronide and sulfate conjugate toxicity, drug-drug interactions (DDIs), pharmacogenetics and species differences are also discussed.

## 2 UGTs, SULTs and Transporters in Human Tissues

**FIGURE 1:**
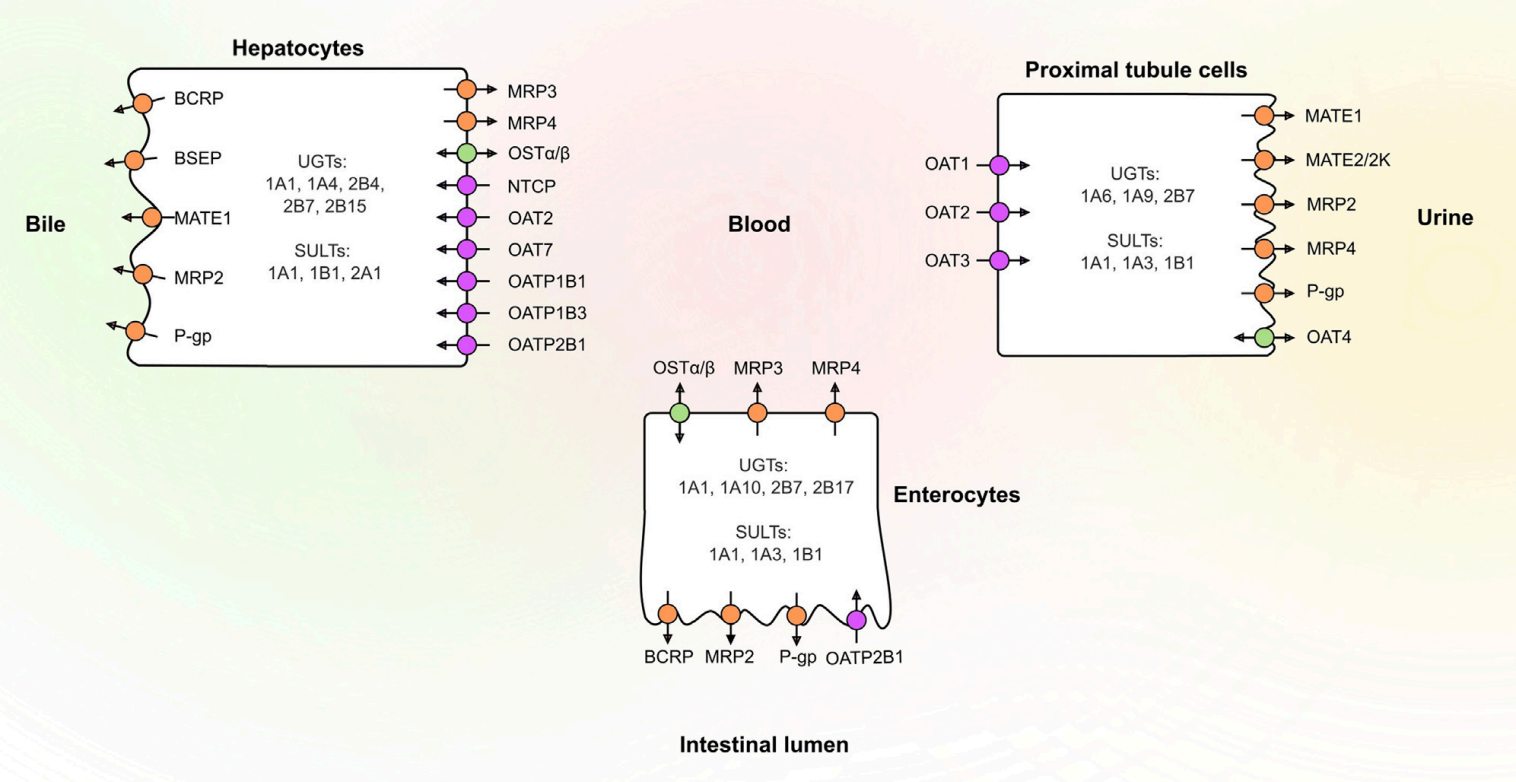
Localization of the main drug transporters in the human liver, kidney and intestine as well as the main phase II enzymes involved in glucuronidation and sulfation reactions of drugs. Uptake transporters are colored in purple, efflux transporters in orange, and bidirectional transporters in green. BCRP, breast cancer resistance protein; BSEP, bile salt export pump; MATE, multidrug and toxin extrusion protein; MRP, multidrug-resistance associated protein; NTCP, sodium/taurocholate cotransporting polypeptide; OAT, organic anion transporter; OATP, organic anion transporting polypeptide; OSTα/β, organic solute transporter α/β; P-gp, P-glycoprotein; SULT, sulfotransferase; UGT, uridine 5′-diphospho-glucuronosyltransferase.

Several UGT and SULT isoforms (e.g. UGT1A1, UGT1A10, UGT2B7, SULT1A1 and SULT1B1) are involved in drug conjugation in human tissues (Oda et al., 2015; Coughtrie, 2016; Basit et al., 2020) (Figure 1). Once formed, these conjugates rely on transporters to cross cell membranes. Transporters are typically divided into two families: Members of the solute carrier (SLC) transporter family function mainly as uptake transporters, whereas ATP-binding cassette (ABC) transporters are primarily efflux transporters (Figure 1). Several members of both families are recognized to play a role in drug disposition and may affect drug safety (Giacomini et al., 2010; Hillgren et al., 2013). These include efflux transporters breast cancer resistance protein (BCRP, *ABCG2*), P-glycoprotein (P-gp, *ABCB1*), multidrug and toxin extrusion proteins (MATEs) and multidrug-resistance associated proteins (MRPs) as well as uptake transporters organic anion transporting polypeptides (OATPs), organic anion transporters (OATs) and organic cation transporters (OCTs). The effects of these transporters on drug and conjugate disposition is dependent on the direction of transport and their polarized localization especially in tissues where metabolism takes place.

### 2.1 Expression in the Intestine

Intestinal metabolism can markedly decrease the bioavailability of drugs. For instance, the bioavailability of testosterone is limited by intestinal UGT2B17 (Zhang et al., 2018). Other major intestinal UGTs are UGT1A1, UGT1A10 and UGT2B7 (Sato Y. et al., 2014; Zhang et al., 2020). The presence of UGT1A10 in the intestine is notable, since it is absent from the human liver and kidney, but can still contribute significantly to glucuronidation (Cubitt et al., 2011; Troberg et al., 2017). The three main SULTs in the intestine are SULT1A1, SULT1A3 and SULT1B1 (Riches et al., 2009). The uptake transporter that is most likely to affect conjugate absorption is OATP2B1 (*SLCO2B1*). OATP2B1 is expressed on the apical membrane of enterocytes, but there is controversy on its localization, as some studies suggest basolateral expression (Keiser et al., 2017). The main efflux transporters in the intestine are BCRP, MRP2 (*ABCC2*), MRP3 (*ABCC3*), MRP4 (*ABCC4*) and P-gp (Drozdzik et al., 2019; Harwood et al., 2019). BCRP, MRP2 and P-gp are expressed at the apical membrane, whereas MRP3 is located at the basolateral membrane of enterocytes. MRP4 appears to show basolateral expression alongside MRP3 (Ming and Thakker, 2010). The relative transport rates of conjugates by basolateral and apical transporters determine the fraction of intestinally formed conjugates that reaches the portal vein or is pumped back to intestinal lumen, respectively. These rates may vary along the intestine, with P-gp and BCRP levels increasing towards the distal end of the small intestine, whereas the levels of MRP2, MRP3, and OATP2B1 are similar in different intestinal sections (Drozdzik et al., 2019).

### 2.2 Expression in the Liver

The liver is a major site of biotransformation and excretion of drugs. Uptake of circulating compounds from the blood into the liver is facilitated on the basolateral (sinusoidal) membranes of hepatocytes by OATP1B1 (*SLCO1B1*), OATP1B3 (*SLCO1B3)* and OATP2B1, sodium/taurocholate cotransporting polypeptide (NTCP*, SLC10A1),* OAT2 (*SLC22A7*), OAT7 (*SLC22A9*) and OCT1 (*SLC22A1*). In the liver, the most abundant UGT is UGT2B7, followed by UGT1A1, UGT1A4, UGT2B4 and UGT2B15 (Fallon et al., 2013; Sato Y. et al., 2014). UGT1A4, and the less abundant UGT2B10, are particularly significant hepatic enzymes, because, unlike most other UGTs, they can catalyze N-glucuronidation of amines. The two main SULTS in the liver are SULT1A1 and SULT2A1 (Riches et al., 2009; Ladumor et al., 2019). Compounds transported into the liver or formed by hepatic metabolism can be effluxed either into blood or excreted into bile. Biliary excretion enables enterohepatic recycling where glucuronide conjugates excreted into bile can be deconjugated by intestinal bacteria and subsequently reabsorbed. On the basolateral membrane of hepatocytes, efflux is mediated by MRP3 and MRP4, whereas biliary excretion is mediated by BCRP, bile salt export pump (BSEP, *ABCB11*), MRP2, P-gp and MATE1 (S*LC47A1*) (Giacomini et al., 2010; Hillgren et al., 2013). BSEP, MRP2, MRP3 and P-gp are found at high levels in the liver, while BCRP and MRP4 abundances are typically low in healthy livers (Burt et al., 2016; Kurzawski et al., 2019; Vildhede et al., 2020). However, MRP3 and MRP4 levels have been shown to increase in several liver diseases (Drozdzik et al., 2020; Vildhede et al., 2020), possibly as an alternative efflux route in hepatocytes in the case of dysfunctional MRP2. MRP3/MRP4 efflux is also a likely prerequisite for hydrophilic conjugates, such as diclofenac-AG, cabotegavir-G and sulfonyloxyaristolactam, formed in the liver, to reach the blood circulation and be excreted into urine (Zhang et al., 2016; Chang et al., 2017; Patel et al., 2019). The interplay between basolateral efflux and uptake transporters in the liver results in a phenomenon called hepatocyte hopping, where transporter substrates are shuttled back and forth between the sinusoidal blood and hepatocytes along the sinusoids (Iusuf et al., 2012). This co-operation between transporters could promote the excretion of various compounds by distributing them between hepatocytes and preventing the saturation of metabolism and canalicular efflux. Hepatocyte hopping is thought to protect hepatocytes from the accumulation of metabolites produced in the liver and is important for endogenous toxins such bilirubin and its glucuronides (Iusuf et al., 2012; Sticova and Jirsa, 2013).

### 2.3 Expression in the Kidney

The kidneys excrete drugs and drug metabolites, in particular hydrophilic conjugates, both through glomerular filtration and transporter-mediated secretion. Conjugates can either be transported into the proximal tubule cells by renal uptake transporters or in some cases formed in the kidney. UGT1A6, UGT1A9 and UGT2B7 are the major renal UGTs (Sato Y. et al., 2014). SULTs (primarily SULT1A1, SULT1A3 and SULT1B1) are expressed in the kidney, but the abundance is much lower than in the intestine or liver (Riches et al., 2009). In the kidney, uptake into proximal tubule cells is primarily mediated by OAT1 (*SLC22A6*), OAT3 (*SLC22A8*) and OCT2 (*SLC22A2*) (Prasad et al., 2016; Li et al., 2019b; Oswald et al., 2019). On the apical membranes of the proximal tubules, MATE1, MRP2, MRP4 and P-gp, are responsible for efflux into urine (Prasad et al., 2016). For example, MRP2 and MRP4 participate in the renal excretion of endogenous glucuronide and sulfate conjugates and may also facilitate excretion of drug conjugates such as glucuronides of non-steroidal anti-inflammatory drugs (NSAIDs), cabotegravir-G and mycophenolic acid glucuronide (MPA-G) (Regan et al., 2010; Matsunaga et al., 2014; Järvinen et al., 2018; Li et al., 2019a; Patel et al., 2019). BCRP, MRP3, MATE2/2K (*SLC47A2*) and OAT2 are present only at low levels, but may contribute to active renal secretion (Fallon et al., 2016; Prasad et al., 2016; Li et al., 2019b; Cheung et al., 2019; Oswald et al., 2019). Notably, in contrast to OAT1 and OAT3, OAT4 (*SLC22A11*) is expressed on the apical membrane of the proximal tubules and may mediate reabsorption of organic anions, such as estrone-S, dehydroepiandrosterone-S (DHEAS) and ethinylestradiol-S (Ugele et al., 2008; Han et al., 2010a).

## 3 Transport of Glucuronide and Sulfate Conjugates

The impact of efflux transporters on the disposition of glucuronide and sulfate conjugates has been reviewed previously (Zamek-Gliszczynski et al., 2006), while an in-depth review of conjugates that interact with uptake transporters is missing. A few examples of phase II drug metabolites as substrates for hepatic and renal uptake transporters have previously been discussed (Zamek-Gliszczynski et al., 2014; Patel et al., 2016). Here we provide an updated comprehensive review of both uptake and efflux transporter interactions with sulfate and glucuronide conjugates. We searched SciFinder and Pubmed databases for literature reports on *in vitro* data on uptake and efflux transporter interactions with glucuronide and sulfate conjugates of drugs and other compounds from studies in transporter overexpression systems. For efflux transporters, the search was limited to articles published after 2006 as earlier data has been compiled by Zamek-Gliszczynski et al. (2006). The complete results of our search, including reports on animal transporters, are reported in Supplementary Table S1.

### 3.1 Uptake Transporters in Humans

In the literature search, a high number of drug as well as other xenobiotic or endogenous glucuronides and sulfates were identified as substrates for human organic anion uptake transporters (Table 1, Supplementary Table S1). Hepatic uptake transporters OATP1B1, OATP1B3, OATP2B1 and NTCP, and renal uptake transporters OAT1, OAT3 and OAT4 all transport both glucuronide and sulfate metabolites of small molecule compounds. Other important uptake transporters OAT2, OAT7, OATP1A2 (*SLCO1A2*), OATP4C1 (*SLCO4C1*) and organic solute transporter α/β (OSTα/β, *SLC51A/B*) are not as well characterized in the transport of conjugated drug metabolites and their significance in this context remains to be fully explored (Supplementary Table S1).

**TABLE 1:** Drug glucuronides (-G and -AG) and sulfates (-S) studied as substrates in transporter overexpression systems.

Drug conjugate^a^	Uptake transporters^b^	Efflux transporters^b^	References
Acetaminophen-G		MRP3	Chloupková et al. (2007)
Brexanolone-S	NTCP		Abu-Hayyeh et al. (2010)
Cabotegravir-G	OAT3	MRP2	Patel et al. (2019)
	OATP1B1	MRP3	
	OATP1B3	MRP4	
	OAT1 (-)	BCRP (-)	
		P-gp (-)	
Cabozantinib M2a (sulfate)	OAT3	MRP2	Lacy et al. (2015)
	OATP1B1	BSEP (-)	
	OATP1B3	P-gp (-)	
	OAT1 (-)		
	OCT1 (-)		
	OCT2 (-)		
Clopidogrel-AG		MRP3	Ji et al. (2018)
Diclofenac-AG	OAT1	BCRP	Zhang et al. (2016); Scialis et al. (2019); Huo et al. (2020)
	OAT2	MRP2	
	OAT3	MRP3	
	OAT4		
	OATP1B1		
	OATP2B1		
	OATP1B3 (-)		
Dihydrotestosterone-G	OATP1B1	BCRP	Li et al. (2019a); Li et al. (2020)
	OATP1B3	MRP2	
	OATP2B1 (-)	MRP3	
		MRP4 (-)	
		P-gp (-)	
E3040-G	OATP1B1	BCRP	Suzuki et al. (2003)
		MRP2	
		MRP3	
		MRP4 (-)	
Edaravone-G		MRP4	Mizuno et al. (2007b)
		BCRP (-)	
		MRP2 (-)	
Edaravone-S	OAT1	BCRP	Mizuno et al. (2007a); Mizuno et al. (2007b)
	OAT3	MRP4 (-)	
Epacadostat-G	OATP1B1	BCRP	Zhang et al. (2017)
	OATP1B3	MRP2	
		MRP3	
Ethinylestradiol-3-G		MRP2	Chu et al. (2004); Chloupková et al. (2007)
		MRP3	
		MRP1 (-)	
Ethinylestradiol-3-S	OAT3	BCRP	Han et al. (2010a); Han et al. (2010b)
	OAT4	BSEP (-)	
	OATP1B1	MATE1 (-)	
	OATP2B1	MRP1 (-)	
	OAT1 (-)	MRP2 (-)	
	OATP1B3 (-)	MRP3 (-)	
	OCT1 (-)	MRP4 (-)	
	OCT2 (-)	P-gp (-)	
Ezetimibe-G	OATP1B1	MRP2	Oswald et al. (2008); Fahrmayr et al. (2012)
	OATP1B3		
	OATP2B1 (-)		
Fasiglifam-AG		MRP2	Kogame et al. (2019)
Fimasartan-G		BCRP	Jeong et al. (2015)
		P-gp	
		MRP1 (-)	
		MRP2 (-)	
Gaboxadol-*O*-G		MRP4	Chu et al. (2009)
		MRP2 (-)	
Gemfibrozil-AG	OATP1B1	MRP2	Hirouchi et al. (2009); Kimoto et al. (2015)
	OATP1B3	MRP3	
	OATP2B1	MRP4	
6-Hydroxymelatonin-S	OAT3		Huo et al. (2017)
	OAT1 (-)OCT2 (-)		
4-Methylumbelliferone-G		BCRP	Järvinen et al. (2017)
		MRP2	
		MRP3	
		MRP4	
(*R*)-Morinidazole-G	OAT3		Zhong et al. (2014)
	OAT1 (-)OCT2 (-)		
(*S*)-Morinidazole-G	OAT3		Zhong et al. (2014)
	OAT1 (-)OCT2 (-)		
Morinidazole-S	OAT1		Zhong et al. (2014)
	OAT3		
	OAT1 (-)OCT2 (-)		
Morphine-3-G		MRP1	van de Wetering et al. (2007)
		MRP2	
		MRP3	
Morphine-6-G		MRP1	van de Wetering et al. (2007)
		MRP2 (-)	
Mycophenolic acid-AG	OATP1B1	MRP2 (-)	Michelon et al. (2010); Patel et al. (2013)
Mycophenolic acid phenyl-G (MPA-G)	OAT3	MRP2	Uwai et al. (2007); Michelon et al. (2010); Picard et al. (2010); Patel et al. (2013); El-Sheikh et al. (2014); Matsunaga et al. (2014); Berthier et al. (2021)
	OATP1B1	MRP3	
	OATP1B3	MRP4	
	OAT1 (-)	MRP8 (-)	
Paroxetine M1-G		BCRP (-)	Matsunaga et al. (2013)
		BSEP (-)	
		MRP2 (-)	
Paroxetine M1-S		BCRP (-)	Matsunaga et al. (2013)
		BSEP (-)	
		MRP2 (-)	
PKI166-G	OATP1B1	MRP2	Takada et al. (2004)
(*R*)-Propranolol-G		MRP3	Järvinen et al. (2017)
		BCRP (-)	
		MRP2 (-)	
		MRP4 (-)	
(*S*)-Propranolol-G		BCRP	Järvinen et al. (2017)
		MRP3	
		MRP4	
		MRP2 (-)	
Raloxifene-4′-G		MRP2	Trdan Lušin et al. (2012a); Kosaka et al. (2015)
		MRP3	
		BCRP (-)	
		MRP1 (-)	
		P-gp (-)	
Raloxifene-6,4′-diG		MRP1	Trdan Lušin et al. (2012a)
		P-gp	
		BCRP (-)	
		MRP2 (-)	
		MRP3 (-)	
Raloxifene-6-G		MRP3	Trdan Lušin et al. (2012a)
		BCRP (-)	
		MRP1 (-)	
		MRP2 (-)	
		P-gp (-)	
Relebactam (sulfate)	OAT3	MATE1	Chan et al. (2019)
	OAT4	MATE2K	
	OAT1 (-)	BCRP (-)	
	OCT2 (-)	MRP2 (-)	
		MRP4 (-)	
		P-gp (-)	
S8921-G	OATP1B1	MRP2	Sakamoto et al. (2008)
	OATP1B3		
	NTCP		
Sorafenib-G	OATP1B1	MRP2	Zimmerman et al. (2013); Vasilyeva et al. (2015); Bins et al. (2017)
	OATP1B3	MRP3	
		MRP4	
Telmisartan-AG	OATP1B3	BCRP	Ishiguro et al. (2008)
	OATP2B1	MRP2	
		P-gp	
Testosterone-G	OATP1B1	MRP2	Li et al. (2019a); Järvinen et al. (2020); Li et al. (2020)
	OATP1B3	MRP3	
	OATP2B1 (-)	BCRP (-)	
		MRP4 (-)	
		P-gp (-)	
Thienorpine-G		MRP2	Kong et al. (2016)
		BCRP (-)	
		P-gp (-)	
Thyroxine-S	NTCP		van der Deure et al. (2008a); Visser et al. (2010)
	OATP1B1		
Triiodothyronine-S	NTCP		van der Deure et al. (2008a); Visser et al. (2010)
	OATP1B1		
Troglitazone-G	OATP1B1	MRP2	Hirouchi et al. (2009)
		MRP3	
		MRP4 (-)	
Troglitazone-S	OATP1B1	BCRP	Nozawa et al. (2004); Enokizono et al. (2007)
	OATP1B3		
	OATP2B1 (-)		
Ursodeoxycholate-AG	OATP1B1		Zhou et al. (2019)
	OATP1B3		
	NTCP (-)		

a The list includes all drug conjugates identified in our literature search, which was limited to years 2007–2021 for efflux transporters. For compounds where data is available for uptake transporters, efflux data is included even if it was published before 2007.

b (-) denotes transporters that have been identified in studies not to transport the conjugate in question.

Organic cation uptake transporters, OCTs and OCTNs, do not appear to interact with conjugate metabolites. For example, neither glucuronides of estradiol, glycochenodeoxycholate (GCDCA) or morinidazole, nor sulfates of estrone, ethinyl-estradiol or morinidazole are substrates for OCTs (Han et al., 2010a, 2010b; Zhong et al., 2014; Bi et al., 2019; Neuvonen et al., 2021). Furthermore, several glucuronides, such fevipiprant-G, baicalein-7-G and epacadostat-G, were reported not to inhibit OCTs and MATEs, although uremic toxin indoxyl-G inhibits OCT2 (Xu et al., 2013; Cheung et al., 2017; Zhang et al., 2017; Poller et al., 2019). A few reports found in the literature search investigated glucuronide and sulfate conjugates as substrates or inhibitors for other human transporters, such as sodium-dependent organic anion transporter (SOAT, *SLC10A6*), orphan transporter SLC22A24, apical sodium-dependent bile acid transporter (ASBT, *SLC10A2*) and other OATPs (e.g. OATP1C1 (*SLCO1C1*) and OATP4A1 (*SLCO4A1*)) (Craddock et al., 1998; Tamai et al., 2000; Pizzagalli et al., 2002; Geyer et al., 2007; Sakamoto et al., 2007; van der Deure et al., 2008b; Fietz et al., 2013; Grosser et al., 2015, 2018; Yee et al., 2019).

#### 3.1.1 Hepatic Uptake Transporters OATP1B1, OATP1B3 and OATP2B1

Uptake transporters OATP1B1 and OATP1B3 transport glucuronide conjugates of several drugs and other xenobiotics, such as flavonoids (Table 1, Supplementary Table S1). Both transporters also play a key role in homeostasis of conjugates of endogenous compounds in the liver. Most importantly, bilirubin glucuronides are high affinity substrates for both OATP1B1 and OATB1B3 and thus, these transporters are partly responsible for controlling plasma levels of conjugated bilirubin (König et al., 2000; Cui et al., 2001; van de Steeg et al., 2010, 2012). Other endogenous conjugate substrates for these transporters include bile acid and steroid conjugates, such as ursodeoxycholate-AG, GCDCA-G and GCDCA-S, glycodeoxycholate-G (GDCA-G) and estradiol-17-G (Takehara et al., 2017; Bi et al., 2019; Zhou et al., 2019; Neuvonen et al., 2021). Glucuronides of several drugs, such as ezetimibe, gemfibrozil and sorafenib, are transported by OATP1B1 and OATP1B3, which may contribute to the enterohepatic recycling of these drugs by directing the excretion of metabolites to the bile and feces, instead of excretion into the urine (Oswald et al., 2008; Hirouchi et al., 2009; Zimmerman et al., 2013; Kimoto et al., 2015; Bins et al., 2017)**.** Similarly, metabolites of therapeutically used hormones, including testosterone-G, dihydrotestosterone-G and ethinylestradiol-S are transported by OATP1B1 or OATP1B3 (Han et al., 2010b; Li et al., 2020).

The transport of drug conjugates by OATP2B1 is not as extensively characterized as for OATP1Bs. OATP2B1 is more widely expressed than the OATP1Bs, and it may therefore affect drug disposition also in the intestine and blood vessels of the heart, brain and other tissues (McFeely et al., 2019). In our literature search, only a few drug glucuronides or sulfates were identified as substrates of OATP2B1 (Table 1). These include ethinylestradiol-S, gemfibrozil-AG, telmisartan-AG and diclofenac-AG (Ishiguro et al., 2008; Han et al., 2010b; Kimoto et al., 2015; Zhang et al., 2016). Estrone-S is an excellent substrate for OATP2B1, while this transporter does not transport estradiol-17-G (Tamai et al., 2001; Bi et al., 2019). Within natural compounds, scutellarein-7-G in particular is a good substrate for OATP2B1, and could even be a specific substrate for this transporter as this compound is not transported by other hepatic organic anion transporters (Gao et al., 2012). Similarly, resveratrol-3-G is highly transported by OATP2B1, weakly by OATP1B1 and OATP1B3 but not by OAT2 or NTCP (Bi et al., 2019). Lastly, some drug conjugates that are transported by OATP1B1 and OATP1B3, such as ezetimibe-G and troglitazone-S, are not OATP2B1 substrates (Nozawa et al., 2004; Oswald et al., 2008).

#### 3.1.2 Hepatic Transporters NTCP and OSTα/β

NTCP is primarily a bile acid transporter, but it can also transport several conjugate metabolites with steroid structures. For example, sulfates of estrone, ethinylestradiol, GCDCA and brexanolone are substrates for NTCP (Abu-Hayyeh et al., 2010; Han et al., 2010b; Takehara et al., 2017). Most studies with NTCP, regarding conjugates, have focused on sulfate metabolites and only one glucuronide, chenodeoxycholate-AG (CDCA-AG), is reported to be a substrate for NTCP (Takehara et al., 2017) (Supplementary Table S1). Similarly, the bidirectional bile acid transporter OSTα/β may have a role in the transport of sulfated metabolites. DHEAS, estrone-S and pregnenolone-S are substrates of OSTα/β, but estradiol-17-G is not (Seward et al., 2003; Ballatori et al., 2005; Fang et al., 2010; Malinen et al., 2019).

#### 3.1.3 Hepatic Uptake Transporters OAT2 and OAT7

Only three conjugate substrates of OAT2 were identified in our literature review. DHEAS and diclofenac-AG are weakly transported by OAT2 (Kobayashi et al., 2005; Zhang et al., 2016), while other studies could not identify OAT2-mediated transport of DHEAS (Hotchkiss et al., 2015; Mathialagan et al., 2018). In contrast, estrone-S was identified as a rather good substrate for OAT2 (Kobayashi et al., 2005; Xu et al., 2013; Mathialagan et al., 2018; Bi et al., 2019). Several glucuronides and sulfates of natural and endogenous compounds, such as quercetin-S and resveratrol-G, estradiol-17-G and glucuronides of GCDCA and GDCA are not transported by OAT2 (Wong et al., 2011a, 2012; Xu et al., 2013; Bi et al., 2019; Neuvonen et al., 2021).

Little is known about OAT7-mediated transport of conjugates. The only identified conjugate substrates of OAT7 are DHEAS and estrone-S (Shin et al., 2007; Ahn et al., 2015; Mathialagan et al., 2018). Inhibition studies also indicate low interaction between conjugates and OAT7. For example, acetaminophen-G, 4-methylumbelliferone-G and vincristine-S did not inhibit OAT7, while minoxidil-S, vinblastine-S and 4-methylumbelliferone-S inhibited OAT7 moderately at best (Shin et al., 2007).

#### 3.1.4 Renal Uptake Transporters OAT1, OAT3 and OAT4

OAT1 and OAT3 transport some glucuronide and sulfate conjugates of endogenous compounds and xenobiotics, but only a small number of drug conjugates have been identified as their substrates (Table 1, Supplementary Table S1). Morinidazole-S, edaravone-S and diclofenac-AG are transported by both OAT1 and OAT3 (Mizuno et al., 2007a; Zhong et al., 2014; Zhang et al., 2016; Huo et al., 2020). On the other hand, OAT3, but not OAT1, transports ethinylestradiol-S and glucuronides such as of cabotegravir-G, curcumin-G, genistein-7-G, steviol-G and MPA-G (Uwai et al., 2007; Han et al., 2010a; Wong et al., 2011b; Wang M. et al., 2015; Zhou et al., 2017; Patel et al., 2019). Moreover, OAT3, but not OAT1, transports endogenous sulfates estrone-S and DHEAS (Ueo et al., 2005) and relebactam, which is a drug molecule containing a sulfate group (Chan et al., 2019). Taken together, it appears that OAT3 may have a more significant role in the renal uptake of glucuronide and sulfate conjugates than OAT1.

The transport profile of OAT4 towards glucuronide or sulfate conjugates has been examined only in a few studies. Of drug conjugates, OAT4 transports ethinylestradiol-S, diclofenac-AG and relebactam (Han et al., 2010a; Zhang et al., 2016; Chan et al., 2019). Other sulfate conjugates transported by OAT4 include quercetin-3′-S and uremic toxin indoxyl-S (Enomoto et al., 2003; Wong et al., 2012). In particular, endogenous sulfates DHEAS, 16α-hydroxy-DHEAS and estrone-S are good substrates for OAT4 (Ugele et al., 2008; Schweigmann et al., 2014). Since OAT4 is localized in the apical membranes of proximal tubule cells, this transporter may have a role in the renal reabsorption of sulfate conjugates. Interestingly, an orphan transporter encoded by the *SLC22A24* gene, and highly homologous to OAT4, was recently identified as a potential renal apical reabsorption transporter for glucuronide and sulfate conjugates of steroids (Yee et al., 2019).

### 3.2 Efflux Transporters in Humans

The role of efflux transporters on phase II conjugate disposition in the liver has been reviewed previously (Zamek-Gliszczynski et al., 2006), but the understanding of conjugate transport has increased within the last 15 years. At the time of the previous review, many endogenous and natural compound conjugates were well-characterized substrates of BCRP, MRP2 and MRP3, but only a handful of drug conjugates had been identified as their substrates *in vitro* (Zamek-Gliszczynski et al., 2006). Since then, numerous drug conjugates as well as conjugated natural compounds that are substrates of these and other efflux transporters have emerged and are discussed below.

#### 3.2.1 MRPs

The majority of drug glucuronides reported after 2006 as efflux substrates are transported by MRP2 and MRP3 (Table 1). MRP2 plays an important role in the biliary excretion of many endogenous organic anions, including estradiol-17-G and bilirubin-Gs (Cui et al., 1999; Kamisako et al., 1999). Based on the current literature, MRP2 may also participate in the disposition of several drug glucuronides. Like OATP1Bs, MRP2 transports ezetimibe-G, MPA-G, sorafenib-G and telmisartan-G, and may contribute to their enterohepatic recycling (Ishiguro et al., 2008; Fahrmayr et al., 2012; Patel et al., 2013; Vasilyeva et al., 2015). In line with previous findings (Zamek-Gliszczynski et al., 2006), MRP2 does not appear to be a prominent sulfate conjugate transporter as only a single sulfated drug metabolite (cabozantinib M2a) and two sulfated natural compounds were identified as substrates of MRP2 in our literature review (Table 1, Supplementary Table S1). In addition to drug metabolites, MRP2 is able to transport flavonoid glucuronides present in herbal medicines (e.g. baicalein-7-G, scutellarein-Gs and wogonin-7-G). MRP2 also transports glucuronides and sulfates of resveratrol, which is found in foods, such as grapes, and used as a herbal supplement (Li et al., 2006; Novelle et al., 2015).

MRP3 is an important transporter for many glucuronide conjugates: Altogether 18 drug glucuronides were identified as MRP3 substrates and only one tested drug glucuronide was reported not to be transported (Table 1). MRP3 transports a wide number of sulfated bile acids (Zelcer et al., 2003; Murai et al., 2013), but otherwise the disposition of sulfated xenobiotics by MRP3 is poorly characterized, and we identified only a single sulfate conjugate (sulfonyloxyaristolactam) as an MRP3 substrate (Chang et al., 2017) (Supplementary Table S1). There is a high degree of overlap between substrates of MRP2 and MRP3, but MRP3 is the only transporter identified to transport acetaminophen-G, clopidogrel-AG and (R-)-propranolol-G (Chloupková et al., 2007; Järvinen et al., 2017; Ji et al., 2018). In addition to drug conjugates, MRP3 transports a wide range of glucuronidated endogenous and natural compounds (Supplementary Table S1). Since MRP3 expels its substrates towards the blood, it can facilitate the entry of conjugates into the systemic circulation and increase their plasma concentrations. For instance, MRP3 substrates epacadostat-G, raloxifene-Gs and scutellarein-6-G all have plasma levels an order of magnitude higher than that of their parent compounds (Chen et al., 2006; Sun et al., 2013; Boer et al., 2016).

MRP4 is reported to transport glucuronides as well as several sulfate conjugates. Since the previous review (Zamek-Gliszczynski et al., 2006), eight glucuronidated drug metabolites have been identified as MRP4 substrates, including several substrates shared with MRP2 and MRP3 (e.g. cabotegravir-G, gemfibrozil-AG, MPA-G and sorafenib-G) (Table 1). In addition, MRP4 transports edaravone-G and gaboxadol-*O*-G, neither of which are transported by MRP2 (Mizuno et al., 2007b; Chu et al., 2009). With respect to sulfated drug conjugates, no substrates have been reported for MRP4 *in vitro*. In contrast, several sulfate conjugates of flavonoids (e.g. chrysin-S) are transported by MRP4, and DHEAS is also an MRP4 substrate (Li et al., 2015; Sun et al., 2015; Järvinen et al., 2017; Kanamitsu et al., 2017). The capability of MRP4 to transport various sulfate conjugates suggests that MRP4 might participate in the efflux of other, yet unidentified sulfate metabolites of drugs.

Limited information is available regarding phase II conjugate transport of other MRPs expressed in different tissues. There are few studies on the ability of MRP1 to transport phase II drug conjugates. Morphine-3-G and morphine-6-G have been shown to be transported by MRP1 *in vitro* (van de Wetering et al., 2007). MRP1 also transports wogonin-7-G, estrone-S and estradiol-17-G (Maeno et al., 2009; Wang et al., 2018). On the other hand, no drug sulfate conjugates were reported to be transported by or inhibit MRP1. Additionally, a limited number of studies investigated the involvement of MRP5 (*ABCC5*), MRP6 (*ABCC6*), MRP7 (*ABCC10*), and MRP8 (*ABCC11*) in the transport of various glucuronide and sulfate conjugates (Supplementary Table S1), but only MRP7 was found to transport a phase II conjugate, estradiol-17-G (Malofeeva et al., 2012).

#### 3.2.2 BCRP

BCRP was previously reported to transport both glucuronides and sulfates of 4-methylumbelliferone and E3040 (Zamek-Gliszczynski et al., 2006). Several new drug conjugate substrates have been identified, such as diclofenac-AG, raloxifene-Gs, telmisartan-AG and troglitazone-S (Table 1). Furthermore, numerous flavonoid and endogenous compound glucuronides and sulfates are known to be transported by BCRP (Supplementary Table S1). Compared to MRPs, BCRP appears to have more sulfate conjugate substrates (Supplementary Table S1). Furthermore, whereas estradiol-17-G is a typical *in vitro* probe substrate of MRPs, estrone-S is preferred for BCRP (Elsby et al., 2011; Brouwer et al., 2013; Pedersen et al., 2017). Similarly, 17α-ethinylestradiol-3-S is a substrate of BCRP, but it is not transported by any of the tested MRPs (Han et al., 2010b). BCRP is, however, capable of transporting several glucuronide conjugates of estrogens (Järvinen et al., 2018).

#### 3.2.3 BSEP

BSEP is expressed exclusively in hepatocytes, where it excretes bile acids into the bile canaliculi and maintains the bile flow. Only few drugs (pravastatin, fexofenadine) are known substrates of BSEP (Hirano et al., 2005; Matsushima et al., 2008), and no glucuronide or sulfate conjugated drugs have been reported to be transported by BSEP. However, glycyrrhizin, which is a diglucuronide of enoxolone extracted from licorice root, has been identified as a BSEP substrate (Dong et al., 2018).

#### 3.2.4 P-gp

P-gp has a negatively charged binding pocket, which is thought to repel anionic compounds (Li et al., 2014; Deng et al., 2020) and is therefore an unlikely candidate for glucuronide and sulfate transport. This is supported by a plethora of studies where conjugates were shown not to be transported by P-gp (Table 1). While a few *in vitro* reports suggest that some endogenous compound and drug glucuronides are P-gp substrates, the involvement of P-gp in the disposition of conjugates is likely low (Supplementary Table S1).

#### 3.2.5 MATEs

Similarly to P-gp, the anionic properties of glucuronide and sulfate conjugates are outside the substrate preferences of MATE1 and MATE2K. One sulfate, relebactam, has been shown to be transported by MATE1 and MATE2K, and not by other drug efflux transporters (e.g. BCRP) (Chan et al., 2019). Additionally, estrone-S is transported by MATE2K, but data on MATE1 transport is contradictory (Tanihara et al., 2007; Shen et al., 2016).

## 4 Interplay of Uptake and Efflux Transport in Drug Conjugate Disposition

**FIGURE 2:**
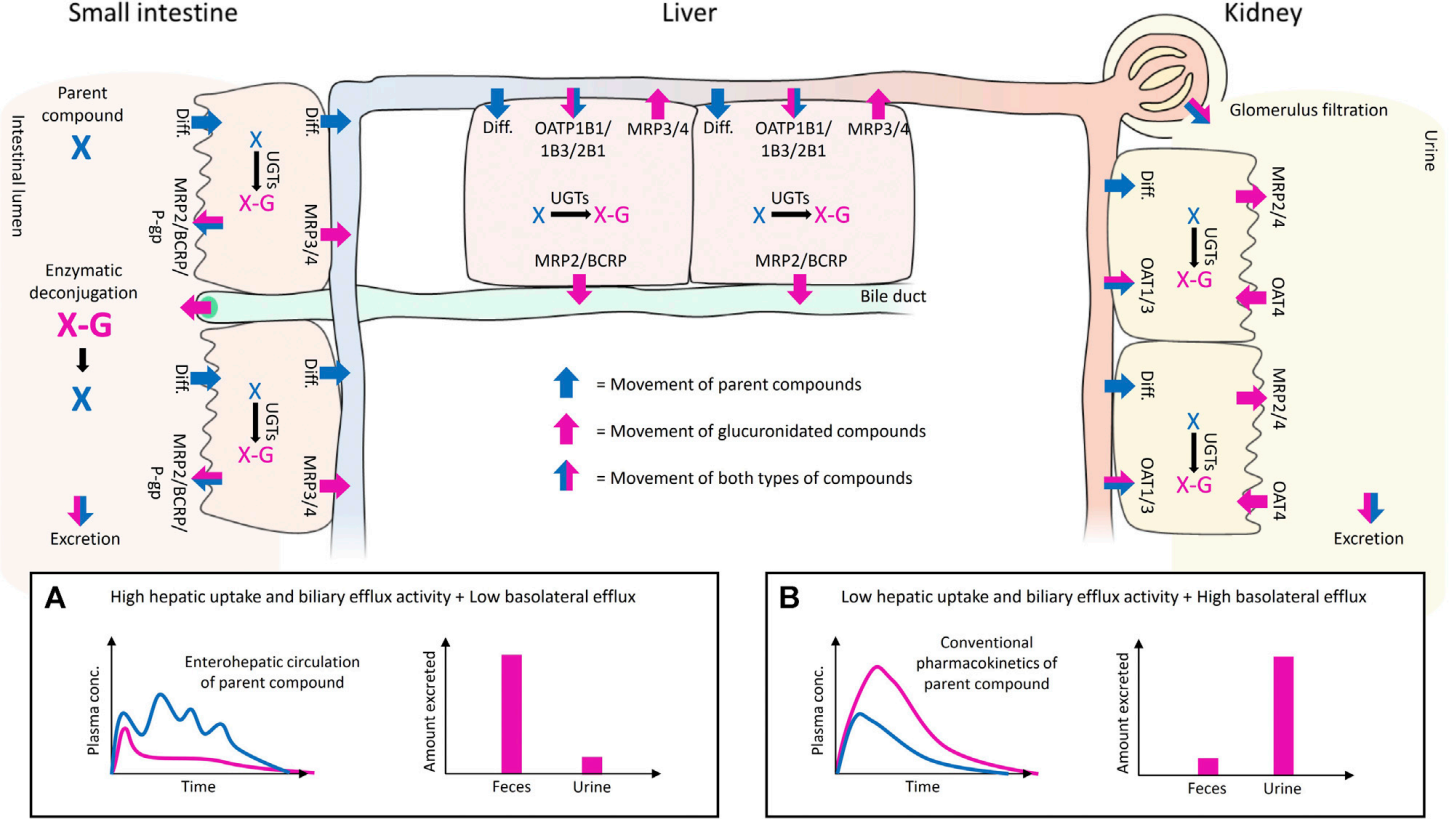
Illustration of the interplay of uptake and efflux transporters in the small intestine, liver, and kidney after oral administration of a parent compound that undergoes glucuronidation. The movement of the parent and its glucuronide conjugate are shown with blue and pink arrows, respectively. The movement of both compounds is shown with half-blue/half-pink arrows. The UGT-mediated glucuronidation of the parent and the enzymatic deconjugation of glucuronidated compounds by bacterial β-D-glucuronidases in the gut are indicated with black arrows. The passive diffusion of compounds over membranes is abbreviated as Diff., and the transporters mentioned in the illustration are considered to be the most relevant transporters for glucuronide disposition in that particular tissue. Examples of uptake and efflux transporter interplay on the disposition of the parent and its glucuronide conjugate are presented in subpanels **(A,B)**. High hepatic uptake and biliary efflux activity, and low hepatic basolateral efflux **(A)** leads to enterohepatic recycling of parent compound (blue line) and favors the fecal excretion of glucuronide conjugate (pink bar). Low hepatic uptake and biliary efflux activity, and high basolateral efflux **(B)** leads to elevated plasma concentration and renal excretion of the glucuronidated compound (pink line and bar).

Phase II metabolism in the liver, intestine and kidney can greatly affect drug exposure. The systemic exposure of conjugated metabolites depends on the metabolic clearance of a parent drug in tissues and the interplay of different transporters expressed on the basolateral and apical membranes of cells (Figure 1 and Figure 2). For instance, some conjugates formed in the intestine may reach the systemic circulation only at low levels if they have high net transport into the intestinal lumen from enterocytes or into hepatocytes over the hepatic basolateral membrane (i.e. high uptake and low efflux). Moreover, high net transport into hepatocytes combined with high biliary efflux typically leads to biliary excretion of glucuronides, which contributes to enterohepatic recycling and prolonged half-life of the parent compound (Figure 2A). In contrast, low hepatic uptake and biliary efflux, together with high basolateral efflux, leads to glucuronide levels that may exceed the levels of the parent drug in plasma, and typically result in urinary excretion of the drug conjugate (Figure 2B). Using four examples, below we highlight how consideration of the interplay of uptake and efflux transporters in conjugate transport can help to explain the observed pharmacokinetic properties of drugs.

### 4.1 Sorafenib-G

Sorafenib is a tyrosine kinase inhibitor that is primarily metabolized by CYP3A4 to sorafenib N-oxide and by UGT1A9 to sorafenib-G (Food and Drug Administration, 2005; Lathia et al., 2006). However, sorafenib exposure was unaltered by ketoconazole in humans, indicating a minor role for oxidative metabolism in its clearance. Approximately 15% of orally administered sorafenib is excreted as sorafenib-G in urine in humans, but most of the dose (77%) is recovered in feces primarily as unchanged drug (50%). Enterohepatic recycling contributes to the pharmacokinetics and long half-life (25–48 h) of sorafenib. Sorafenib-G is a substrate for human MRP2, MRP3 and MRP4 (Table 1), which means that it can both be effluxed into the blood and undergo biliary excretion in the liver, as demonstrated in sandwich-cultured human hepatocytes (Swift et al., 2013). Sorafenib-G is also a substrate of OATP1B1 and OATP1B3 (Table 1), which together with MRP3-mediated hepatocyte hopping may explain the low levels of sorafenib-G found in human plasma (European Medicines Agency, 2006; Vasilyeva et al., 2015). Moreover, UGT1A9 is highly expressed in the kidney and it is likely that direct renal glucuronidation may contribute to the urinary excretion of sorafenib-G. The low levels of sorafenib-G in feces may be explained by bacteria-mediated deconjugation of the conjugate in the intestine. This is supported by a 54% reduction in sorafenib area under the concentration time curve (AUC) after administration of the antibiotic neomycin in humans (European Medicines Agency, 2006), which also highlights the role of sorafenib-G in the enterohepatic recycling and prolonged exposure of sorafenib.

### 4.2 Raloxifene-Gs

Raloxifene is a selective oestrogen receptor modulator indicated for osteoporosis treatment and prevention in postmenopausal women. It has low absolute bioavailability (2%) due to extensive first-pass glucuronidation and most of the raloxifene in human plasma is in the form of raloxifene-4′-G, raloxifene-6-G and raloxifene-6,4′-di-G (Hochner-Celnikier, 1999; Snyder et al., 2000; Trdan Lušin et al., 2012b). Unconjugated raloxifene represents less than 1% of total drug material in human plasma. The half-life of raloxifene is long (30 h) and secondary plasma peaks of raloxifene appear in human plasma indicating enterohepatic recycling. The main excretion pathway of raloxifene and its glucuronides is in feces and less than 6% of the dose is excreted as glucuronides in urine. Intestinal metabolism contributes significantly to raloxifene clearance and even exceeds the clearance measured in human liver microsomes *in vitro* (Gufford et al., 2015). From enterocytes, raloxifene-4′-G and raloxifene-6-G can reach the portal circulation via MRP3-mediated transport, whereas MRP2 could efflux raloxifene-4′-G to the intestinal lumen (Trdan Lušin et al., 2012a; Kosaka et al., 2015). No reports on uptake transport of raloxifene glucuronides are available, but raloxifene-4′G and raloxifene-6,4′-di-G strongly inhibit OATP1B1 and OATP1B3 (Table 2), showing that these glucuronides interact with OATPs, which may mediate their hepatic uptake. In the liver, raloxifene-Gs are likely to be excreted into bile by MRP2. While MRP3 can efflux raloxifene-Gs into blood from hepatocytes, the high recovery of administered raloxifene in feces supports predominant biliary excretion as a consequence of high MRP2 transport and high net uptake into hepatocytes, likely mediated by OATPs. Although raloxifene undergoes sulfate conjugation in human enterocytes, intestinal microsomes and liver microsomes, raloxifene sulfates were not detected in human plasma (Food and Drug Administration, 1999; Hui et al., 2015; Davies et al., 2020). The systemic absorption of raloxifene sulfates may be limited by apical efflux transporters (Jeong et al., 2004; Zhou et al., 2015).

**TABLE 2:** Drug glucuronides (-G and -AG) and sulfates (-S) studied as inhibitors in transporter overexpression systems.

Drug conjugate	Inhibited uptake transporter^a^	Inhibited efflux transporter^a^	References
Abiraterone-*N*-oxide-S	OAT3 (K_i_ = 1 µM)		Zou et al. (2021)
	OAT1 (-)		
Abiraterone-S	OAT1 (K_i_ = 38 µM)		Zou et al. (2021)
	OAT3 (K_i_ = 2 µM)		
Brexanolone-S	NTCP (K_i_ = 8 µM)		Abu-Hayyeh et al. (2010)
Cabozantinib M2a (sulfate)	OAT1 (IC_50_ = 4 µM)	BSEP (IC_50_ = 50 µM)	Lacy et al. (2015)
	OAT3 (IC_50_ = 4 µM)	MATE1 (IC_50_ = 17 µM)	
	OATP1B1 (IC_50_ = 6 µM)	MATE2K (IC_50_ = 65 µM)	
	OATP1B3 (IC_50_ = 21 µM)	MRP2 (IC_50_ = 79 µM)	
	OCT1 (-)	P-gp (-)	
	OCT2 (-)		
Clopidogrel-AG	OATP1B1 (IC_50_ = 11-51 µM)		Tamraz et al. (2013); Shebley et al. (2017)
Diclofenac-AG	OAT1 (IC_50_ = 265 µM)	BCRP (20% at 100 µM)	Nozaki et al. (2007); Kawase et al. (2016); Iwaki et al. (2017)
	OAT3 (IC_50_ = 3 µM)	MRP2 (IC_50_ = 19 µM)	
		MRP4 (IC_50_ = 140 µM)	
Epacadostat-G	OATP1B1 (IC_50_ = 262 µM)	BCRP (-)	Zhang et al. (2017)
	OATP1B3 (IC_50_ = 27 µM)	P-gp (-)	
	OAT1 (-)		
	OAT3 (-)OCT2 (-)		
Ezetimibe-G	OATP1B1 (IC_50_ = 0.2 µM)	BCRP (IC_50_ = 52 µM)	Oswald et al. (2006); Oswald et al. (2008); de Waart et al. (2009)
	OATP1B3 (IC_50_ = 0.3 µM)	MRP2 (IC_50_ = 34 µM)	
	OATP2B1 (IC_50_ = 0.1 µM)	MRP3 (IC_50_ = 7 µM)	
		P-gp (60% at 100 µM)	
Fasiglifam-AG	OATP1B1 (IC_50_ = 1 µM)	BSEP (IC_50_ = 33 µM)	Otieno et al. (2018); Ackerson et al. (2019)
	OATP1B3 (IC_50_ = 1 µM)	MRP2 (IC_50_ = 1 µM)	
		MRP3 (IC_50_ = 0.2 µM)	
		MRP4 (IC_50_ = 0.9 µM)	
Fevipiprant-AG	OAT3 (K_i_ = 16 µM)		Poller et al. (2019)
	OATP1B1 (K_i_ = 31 µM)		
	OATP1B3 (K_i_ = 12 µM)		
	OAT1 (-)		
(*R*)-Flurbiprofen-AG	OAT1 (IC_50_ =198 µM)	MRP2 (IC_50_ = 30 µM)	Kawase et al. (2016); Iwaki et al. (2017)
	OAT3 (IC_50_ = 19 µM)	MRP4 (IC_50_ = 3 µM)	
(*S*)-Flurbiprofen -AG	OAT1 (IC_50_ = 174 µM)	MRP2 (IC_50_ = 22 µM)	Kawase et al. (2016); Iwaki et al. (2017)
	OAT3 (IC_50_ = 32 µM)	MRP4 (IC_50_ = 93 µM)	
Gemfibrozil-AG	OAT3 (IC_50_ = 20 µM)		Hirano et al. (2006); Nakagomi-Hagihara et al. (2007a, 2007b)
	OATP1B1 (K_i_ = 8-23 µM)		
(*R*)-Ibuprofen -AG	OAT1 (IC_50_ = 791 µM)	MRP2 (IC_50_ = 208 µM)	Kawase et al. (2016); Iwaki et al. (2017)
	OAT3 (IC_50_ = 60 µM)	MRP4 (IC_50_ = 4 µM)	
(*S*)-Ibuprofen-AG	OAT1 (IC_50_ = 960 µM)	MRP2 (IC_50_ = 81 µM)	Kawase et al. (2016); Iwaki et al. (2017)
	OAT3 (IC_50_ = 57 µM)	MRP4 (IC_50_ = 67 µM)	
(*S*)-Ketoprofen-AG	OAT1 (K_i_ = 40 µM)		Zou et al. (2021)
	OAT3 (K_i_ = 8 µM)		
Micafungin (sulfate)		BCRP (IC_50_ = 21 µM)	Lempers et al. (2016)
		BSEP (IC_50_ = 85 µM)	
		MRP1 (IC_50_ = 21 µM)	
		MRP2 (IC_50_ = 148 µM)	
		MRP3 (IC_50_ = 42 µM)	
		MRP4 (IC_50_ = 4 µM)	
		MRP5 (IC_50_ = 22 µM)	
		P-gp (IC_50_ = 45 µM)	
MK-8666-AG		BSEP (28% at 25 µM)	Hafey et al. (2020)
Mycophenolic acid-AG	OAT1 (13% at 100 µM)		Wolff et al. (2007)
	OAT3 (IC_50_ = 3 µM)		
Mycophenolic acid phenyl-G (MPA-G)	OAT1 (IC_50_ = 223-512 µM)	MRP2 (IC_50_ = 1037 µM)	Takekuma et al. (2007); Uwai et al. (2007); Wolff et al. (2007)
	OAT3 (IC_50_ = 15-69 µM)		
(*R*)-Naproxen-AG	OAT1 (IC_50_ = 639 µM)	MRP2 (IC_50_ = 771 µM)	Kawase et al. (2016); Iwaki et al. (2017)
	OAT3 (IC_50_ = 129 µM)	MRP4 (IC_50_ = 2 µM)	
(*S*)-Naproxen-AG	OAT1 (IC_50_ = 747 µM)	BCRP (20% stimulation at 100 µM)	Nozaki et al. (2007); Kawase et al. (2016); Iwaki et al. (2017); Zou et al. (2021)
	OAT3 (K_i_ = 5 µM)	MRP2 (IC_50_ = 475 µM)	
		MRP4 (IC_50_ = 49 µM)	
Probenecid-AG	OAT1 (K_i_ = 130 µM)		Zou et al. (2021)
	OAT3 (K_i_ = 20 µM)		
Raloxifene-4′-G	OATP1B1 (65% at 10 µM) OATP1B3 (100% at 10 µM)	BCRP (IC_50_ = 0.3 µM)	Trdan Lušin et al. (2012a); Trdan Lušin et al. (2012b)
		MRP1 (IC_50_ = 4 µM)	
		MRP2 (IC_50_ = 2 µM)	
		MRP3 (IC_50_ = 8 µM)	
		P-gp (IC_50_ = 6 µM)	
		BSEP (-)	
Raloxifene-6,4′-diG	OATP1B1 (54% at 4 µM) OATP1B3 (100% at 4 µM)	BCRP (IC_50_ = 3 µM)	Trdan Lušin et al. (2012a); Trdan Lušin et al. (2012b)
		MRP1 (IC_50_ = 2 µM)	
		MRP2 (50% at 4 µM)	
		MRP3 (IC_50_ = 0.5 µM)	
		P-gp (IC_50_ = 0.8 µM)	
		BSEP (-)	
Raloxifene-6-G	OATP1B1 (-)	BCRP (IC_50_ = 40 µM)	Trdan Lušin et al. (2012a); Trdan Lušin et al. (2012b)
	OATP1B3 (-)	MRP1 (IC_50_ = 1 µM)	
		MRP3 (IC_50_ = 10 µM)	
		P-gp (IC_50_ = 10 µM)	
		BSEP (-)	
		MRP2 (-)	
Relebactam (sulfate)	OAT1 (-)	BSEP (12% at 500 µM)	Chan et al. (2019)
	OAT3 (-)	P-gp (16% at 300 µM)	
	OATP1B1 (-)	BCRP (-)	
	OATP1B3 (-)	MATE1 (-)	
	OCT2 (-)	MATE2K (-)	
Rosiglitazone-5-hydroxy-S	OAT1 (K_i_ = 34 µM)		Zou et al. (2021)
	OAT3 (K_i_ = 1 µM)		
SN-38-G	OATP1B1 (13% at 10 µM)		Nozawa et al. (2005)
Thyroxine-G	OATP2B1 (IC_50_ = 45 µM)		Meyer Zu Schwabedissen et al. (2018)
Tolmetin-AG	OAT1 (K_i_ = 7 µM)		Zou et al. (2021)
	OAT3 (K_i_ = 3 µM)		
Troglitazone-G	OATP1B1 (69% at 10 µM)		Nozawa et al. (2004)
	OATP1B3 (12% at 10 µM)		
Troglitazone-S	OATP1B1 (95% at 10 µM)	MRP4 (K_i_ = 8 µM)	Nozawa et al. (2004); Yang et al. (2015); Malinen et al. (2019)
	OATP1B3 (83% at 10 µM)		
	OSTα/β (IC_50_ = 191 µM)		
Vericiguat-G	OATP1B1 (IC_50_ = 26 µM)	MATE1 (-)	Boettcher et al. (2020)
	OATP1B3 (IC_50_ = 17 µM)	MATE2K (-)	
		P-gp (-)	

a Inhibition reported as inhibitory constant (K_i_), half-maximal inhibitory concentration (IC_50_) or inhibition percentage at a defined concentration of the inhibitor. (-) denotes transporters that have been shown not to be inhibited.

### 4.3 Epacadostat-G

Epacadostat is an indoleamine 2,3-dioxygenase inhibitor being developed for cancer treatment. Its most abundant metabolite is epacadostat-G, which is formed by UGT1A9 in the liver. At steady state, epacadostat-G is found at 8-fold levels compared to epacadostat in human plasma, which can be explained by efficient basolateral efflux by MRP3 (Boer et al., 2016; Zhang et al., 2017). Epacadostat exhibits a pharmacokinetic profile with double peaking that indicates enterohepatic recycling of the drug. Epacadostat-G is likely involved in this recycling as it is excreted into bile by MRP2 and BCRP and hydrolyzed completely in incubations with human feces (Boer et al., 2016; Zhang et al., 2017). Epacadostat-G is also a substrate of OATP1B1 and OATP1B3, which may enhance its biliary excretion and contribution to the enterohepatic recycling of epacadostat (Zhang et al., 2017). The renal clearance of epacadostat and its metabolites is minimal at least in preclinical species (Zhang et al., 2017), but human excretion data is not available.

### 4.4 Cabotegravir-G

Cabotegravir is a newly approved integrase strand transfer inhibitor for HIV treatment, which is glucuronidated primarily in the liver by UGT1A1 and UGT1A9 to form cabotegravir-G (Bowers et al., 2016). Cabotegravir-G is a substrate of MRP2, and cabotegravir-G was found in the bile of some human subjects, whereas the parent drug was found in all bile samples in line with its primary excretion in feces (47%) in humans (Bowers et al., 2016; Patel et al., 2019). Cabotegravir-G also undergoes sinusoidal efflux by MRP3 and MRP4 (Patel et al., 2019). Approximately 20% of the oral dose is recovered in urine as cabotegravir-G, but cabotegravir-G levels in the systemic circulation are negligible (Bowers et al., 2016). This behavior is explained by efficient renal elimination of cabotegravir-G that is mediated by OAT3 on the apical and MRP2 and MRP4 on the basolateral membranes of renal proximal tubule cells (Patel et al., 2019). In addition, renal glucuronidation of cabotegravir by UGT1A9 and direct efflux of the metabolite into urine may contribute to the renal excretion of cabotegravir-G.

## 5 Conjugates as Inhibitors of Transporters

Transporters are known to mediate DDIs (Gessner et al., 2019). *In vitro* inhibition studies can identify compounds that affect transporter activity and might cause DDIs or other transporter-mediated toxicity. A list of transporter inhibitors within glucuronide and sulfate conjugates are compiled in Table 2 for drug conjugates and the full list is available in Supplementary Table S1. Among phase II conjugates, some strong inhibitors of hepatic uptake and efflux transporters have been identified. For example, ezetimibe-G, a substrate of OATPs and MRP2, is also a strong inhibitor of OATPs (half maximal inhibitory concentration (IC_50_) <0.5 µM) and can inhibit BCRP, MRP2 and MRP3 (Oswald et al., 2008; de Waart et al., 2009). Another drug conjugate with strong inhibition potential for both OATP1Bs and MRPs is fasiglifam-G, which has IC_50_ values ≤1 µM for these transporters (Otieno et al., 2018; Ackerson et al., 2019). Raloxifene-4′-G and raloxifene-6,4′-di-G appear able to inhibit all of the major hepatic drug transporters with IC_50_ values <10 µM, but interpretation warrants caution as values for the efflux transporters are based on changes in ATPase activity and not inhibition of transport (Trdan Lušin et al., 2012a; 2012b). Interestingly, the third raloxifene glucuronide, raloxifene-6-G, did not inhibit OATP1B1, OATP1B3 or MRP2.

Reports on transporter inhibition by drug sulfates are scarce, but the sulfate conjugate of a metabolite of tyrosine kinase inhibitor cabozantinib (cabozantinib M2a) inhibits efflux transporters BSEP, MATE1, MATE2K and MRP2 with IC_50_ values of 17–79 µM (Lacy et al., 2015). Cabozantinib M2a also inhibits renal and hepatic uptake transporters OAT1, OAT3, OATP1B1 and OATP1B3 with IC_50_ values of 4–21 µM (Lacy et al., 2015). Micafungin, a large antifungal compound which contains a sulfate group, inhibits BCRP, BSEP, P-gp, and several MRPs (Lempers et al., 2016). The strongest inhibition was towards MRP4 with an IC_50_ value of 4 µM. MRP4 and rat Bsep are inhibited by troglitazone-S, which contributes to the hepatotoxicity of troglitazone (Funk et al., 2001; Masubuchi, 2006; Yang et al., 2015). Importantly, despite having a low number of drug substrates, BSEP is susceptible to drug-induced inhibition, which may lead to intrahepatic accumulation of bile acids and drug-induced liver injury (Kenna et al., 2018). Therefore, the inhibitory potential of conjugate metabolites should be considered.

In the kidney, acyl glucuronides of flurbiprofen, ibuprofen and naproxen inhibit OAT3 with IC_50_ values 19–129 µM (Iwaki et al., 2017). Stronger inhibition (IC_50_ <3 µM) was observed by diclofenac-AG, tolmetin-AG and mycophenolic acid-AG (Wolff et al., 2007; Iwaki et al., 2017; Zou et al., 2021). Sulfate conjugates may also strongly inhibit OAT3. Sulfates of abiraterone, abiraterone-N-oxide and 5-hydroxy-rosiglitazone inhibit OAT3 with inhibitory constant (K_i_) values below 2 µM (Zou et al., 2021). Although OAT1 has fewer conjugate substrates than OAT3, conjugated metabolites can inhibit OAT1. Diclofenac-AG, flurbiprofen-AG, naproxen-AG and ibuprofen-AG have IC_50_ values between 174–960 µM towards OAT1. More potent OAT1 inhibitors are abiraterone-S, (*S*)-ketoprofen-AG, rosiglitazone-5-hydroxy-S and tolmetin-AG with K_i_ values between 7–40 µM (Zou et al., 2021).

Many natural compounds found either in foods or herbal supplements are known to inhibit transporters (Supplementary Table S1). BCRP and MRP2, in particular, are inhibited by several glucuronide and sulfate conjugated natural compounds. For example, chrysin-7-G, hesperitin-3′-G, hesperitin-7-G and quercetin-3-G all inhibit BCRP and MRP2 with IC_50_ <50 µM. Quercetin-Gs also inhibit OAT1 and OAT3 with IC_50_ values even below 1 µM (Wong et al., 2011b). Baicalein-7-G is a well-characterized inhibitor of both uptake and efflux transporters, with IC_50_ values <20 µM for BCRP, MRP3, MRP4 OATP1B3, OATP2B1, OAT3 and OAT4 (Xu et al., 2013; Kalapos-Kovács et al., 2015). Scutellarein-7-G (scutellarin) is an inhibitor of OATP2B1 with IC_50_ of 2–5 µM (Wen et al., 2016; Iijima et al., 2018) and it also inhibits BCRP and MRP2 (Gao et al., 2012). Chrysin-7-S is a potent inhibitor of BCRP, OATP1B1 and OATP2B1 (IC_50_ <1 µM) and inhibits MRP2, but not as well as chrysin-7-G (Mohos et al., 2020a). Furthermore, quercetin-3′-S inhibits OATP1B1, OATP2B1, OAT1 and OAT3 with IC_50_ <1 µM (Wong et al., 2011b; Mohos et al., 2020b).

## 6 Clinical Drug Interactions Involving Conjugates

To date, little data is available on clinically significant DDIs, in which conjugates act as victims or perpetrators. Given the role of transporters in drug disposition and the potential of glucuronide and sulfate metabolites to inhibit these transporters *in vitro* (Table 2), clinical DDIs might be mediated by phase II metabolites. Inhibition of conjugate transport, on the other hand, may reduce excretion or enterohepatic recycling of the drug conjugates, which can increase conjugate levels, but decrease exposure of the parent and thus reduce treatment efficacy. DDIs can be of special concern for phase II conjugates as victim drugs for those drugs with potentially serious side effects, such as opioid or kinase inhibitor conjugates (e.g. morphine-6-G and sorafenib-G). Several of the known drug interactions involving drug conjugates (e.g. disruption of enterohepatic recycling of MPA-G by cyclosporine or inhibition of BSEP mediated biliary excretion of bile acids by troglitazone-S) have been reviewed previously (Zamek-Gliszczynski et al., 2014; Patel et al., 2016), but some new findings are discussed below.

### 6.1 Sorafenib-G: Altered Conjugate Disposition due to Transporter Inhibition

As described in Section 4.1, sorafenib-G is excreted into bile by MRP2 and undergoes OATP1B1- and OATP1B3-mediated reuptake into hepatocytes, after basolateral efflux by MRP3, to enhance biliary excretion. When this re-uptake was interrupted with the OATP inhibitor rifampicin (600 mg once daily twice) in healthy volunteers, the systemic exposure to sorafenib-G rose over 2-fold, while there were no significant differences in sorafenib parameters (Bins et al., 2017). Increased sorafenib-G exposure was also observed in Oatp1b2-deficient mice, indicating that OATP inhibition was the mechanism of the observed rifampicin interaction (Bins et al., 2017). This serves as an example of *in vivo* OATP inhibition affecting conjugate disposition. Long-term treatment with rifampicin leads to a decrease in sorafenib AUC by 37% (Food and Drug Administration, 2005), which may be caused by several reasons, such as induction of glucuronidation and hepatic efflux transport. However, many compounds can inhibit OATPs (Karlgren et al., 2012) and not all of them are inducers of metabolic enzymes or transporters. Recently, administration of probenecid with sorafenib was shown to increase the ratio of sorafenib-G to sorafenib in plasma due to a reduction in sorafenib exposure (Hussaarts et al., 2020). This was suggested to be caused by disrupted enterohepatic recycling caused by inhibition of OATP1B1, as with rifampicin, but surprisingly the plasma concentrations of the glucuronide were unaffected.

### 6.2 Clopidogrel-AG: Inhibition of Transport by a Drug Conjugate

Even though conjugate-mediated transporter inhibition might not be the primary mechanistic source of many DDIs, they can be complicit in adding to complex DDIs. Clopidogrel-AG is a mechanism-based inhibitor of CYP2C8, but also a substrate and inhibitor of transporters (Table 1 and Table 2, (Tornio et al., 2014)). Co-administration of clopidogrel increased repaglinide AUC by 5.1-fold (Tornio et al., 2014). While mechanism-based inhibition of CYP2C8 by clopidogrel-AG is the primary contributor to this interaction, OAT1B1 inhibition by clopidogrel and its acyl glucuronide increases the severity of the interaction by 1.5-fold based on pharmacokinetic simulations. Although there are no clinical reports, MRP3 function may also affect this interaction by modulating the intracellular concentrations of clopidogrel-AG. Indeed, the liver-to-plasma ratio of clopidogrel-AG was 11-fold higher in Mrp3 knockout mice compared to wild type, supporting the role of MRP3 in sinusoidal efflux (Ji et al., 2018). This was observed also in a clinical setting, where the plasma AUC of clopidogrel-AG was 1000 times higher than of the parent (Tornio et al., 2014). It is unknown if clopidogrel-AG itself undergoes re-uptake into hepatocytes by uptake transporters from the blood circulation.

### 6.3 Conjugates as Biomarkers of Drug Inhibition

The use of endogenous transporter substrates as biomarkers of transporter function can help to avoid dedicated DDI studies and be used to assess DDI risks of drugs that have ethical and practical limitations when it comes to conventional DDI trials (Chu et al., 2018). Many endogenous conjugates, primarily bile acid conjugates, have been studied for their utility as biomarkers to assess the inhibitory effects of drugs on transporters. Although this may be challenging considering the overlap between transporters, some promising conjugates have been identified, especially for OATP1Bs.

The bile acid conjugate GCDCA-S has been proposed as a biomarker for several transporters. As a biomarker of OAT1/OAT3-mediated DDIs, probenecid reduced the renal clearance of GCDCA-S in a dose-dependent manner. The degree of reduction was similar to changes for the OAT1/OAT3 probe drug benzyl penicillin, even though apically expressed MRP2 in the proximal tubules might also be involved in the decreased GCDCA-S excretion (Tsuruya et al., 2016). GCDCA-S and other bile acid conjugates have also been studied as OATP1B biomarkers for detecting DDIs. A 600 mg single dose of rifampicin increased GCDCA-S AUC over 20-fold (Takehara et al., 2018). A dose-dependent effect of rifampicin on the AUCs of GCDCA-S, GCDCA-G and CDCA-AG was later confirmed (Mori et al., 2020b). The utility of bile acid conjugates as biomarkers for OATP1B-mediated DDIs was additionally investigated with paclitaxel at therapeutic doses in non-small cell lung cancer patients (Mori et al., 2020a). In this study, the AUC of several sulfate and glucuronide conjugates (e.g. GCDCA-S, GCDCA-G, CDCA-AG and GDCA-S) increased over 2.5-fold with paclitaxel administration. It should be noted however, that some of these compounds are also substrates of efflux transporters (Neuvonen et al., 2021), which may complicate interpretation. Clinical studies to identify biomarkers for OATP1B3 have not yet been conducted, but testosterone-G and androsterone-G were recently identified to be primarily transported by OATP1B3 and have been proposed as potential OATP1B3 biomarkers (Li et al., 2020). Plasma levels of DHEAS, a testosterone precursor, appear to be insensitive towards OATP1B inhibition by rifampicin in humans (Shen et al., 2017).

## 7 Conjugate Toxicity Involving Transporter-Mediated Disposition

Some phase II conjugates have been linked to drug toxicity, since molecules containing carboxylic acid moieties can be metabolized into reactive, electrophilic acyl glucuronides and may thus require safety assessment (Food and Drug Administration, 2020). Acyl glucuronides are formed from many widely used drugs (e.g. NSAIDs, mycophenolic acid, valproic acid and gemfibrozil) and they can covalently bind to proteins and DNA, increase oxidative stress or trigger an immune response contributing to the risk of idiosyncratic drug toxicity (Regan et al., 2010). Transporters play a key role in regulating intracellular acyl glucuronide levels and transporter function may therefore influence their toxicity risk. Furthermore, transporters may also affect the toxicity of environmental toxins that form reactive sulfate conjugates (Glatt, 2000).

### 7.1 Fasiglifam-AG

Fasiglifam (TAK-875) is a free fatty acid receptor 1 agonist that was in development for the treatment of type 2 diabetes (Kaku et al., 2015). It is a recent example of a drug with an acyl glucuronide that was withdrawn during phase III clinical trials due to safety concerns regarding drug-induced liver injury (DILI). Formation of fasiglifam-AG is the major metabolic pathway for fasiglifam in humans and *in vitro* studies have indicated that its high risk for DILI is attributable mainly to this metabolite (Kaku, 2013) (Thompson et al., 2012). Fasiglifam-AG showed nonlinear accumulation in rat livers with dose escalation, suggestive of saturation of biliary efflux, likely from inhibition of Mrp2 (Otieno et al., 2018; Kogame et al., 2019). The role of uptake transporters in fasiglifam-AG disposition is unknown, but it is an inhibitor of several hepatic uptake and efflux transporters (Table 2). Based on observed plasma and liver concentrations of fasiglifam-AG in rats, it is likely to inhibit hepatic efflux transporters *in vivo*, but not uptake transporters. Moreover, fasiglifam-AG is a more potent inhibitor of human than rat MRPs/Mrps, as the IC_50_ values were over 10- and 50-fold higher for rat Mrp2 and Mrp4 than for human MRP2 and MRP4, while MRP3 inhibition was more similar (Otieno et al., 2018). In addition to direct fasiglifam-AG-mediated liver toxicity, fasiglifam and fasiglifam-AG can alter bile acid homeostasis by inhibiting MRP2, MRP3, MRP4, NTCP and BSEP, possibly contributing to DILI (Wolenski et al., 2017).

### 7.2 Diclofenac-AG

Diclofenac is used widely (e.g. in the treatment of osteoarthritis) and has been associated with enteropathy, kidney toxicity and rare but severe idiosyncratic hepatotoxicity (Banks et al., 1995; Douros et al., 2018; Watanabe et al., 2020). Histological samples collected from patients having adverse reactions to diclofenac showed hepatocellular injury in over 70% of the samples (Banks et al., 1995). Diclofenac-AG is formed in the liver by UGT2B7 (King et al., 2001) and transporters play a key role in its disposition (Table 1). In the liver, diclofenac-AG is excreted into bile by BCRP and MRP2, whereas MRP3 serves as an alternative pathway if biliary excretion is impaired (Lagas et al., 2010). In humans, diclofenac-AG is primarily excreted in urine. After efflux into the blood by MRP3 in hepatocytes, diclofenac-AG is taken up by OAT1/3 in the kidney and can be transported on the apical side by OAT4 (Zhang et al., 2016). This uptake can lead to renal accumulation and predispose the tubular cells to direct cytotoxicity, which in addition to the reduction in afferent arterial flow caused by diclofenac is considered to be one of the main mechanisms of diclofenac nephrotoxicity. *In vitro*, the toxicity of diclofenac-AG could be reduced by OAT1/3 inhibition by cilastatin (Huo et al., 2020). Diclofenac-AG can increase diclofenac exposure, as acyl glucuronides can be deconjugated non-enzymatically or by esterases both in the liver and plasma (Suzuki et al., 2010; Ito et al., 2014). Due to this conversion, cilastatin increased the AUC of both diclofenac and diclofenac-AG in mice, even though diclofenac itself is not an OAT1/3 substrate (Huo et al., 2020). MRP3 in the intestine may also protect against diclofenac induced enteropathy. Intestinal ulceration is a classic adverse effect of NSAIDs caused by their pharmacological mode of action and the accumulation of AG metabolites can aggravate it. Studies in Mrp3 knockout mice showed that intestinal injuries caused by diclofenac were consistently more severe in knockout compared to wild type mice (Niu et al., 2015). Ulceration was also reduced in Mrp2 knockout rats compared with wild type rats, presumably due to decreased diclofenac-AG biliary clearance (Seitz and Boelsterli, 1998).

### 7.3 Other Toxins

Aristolochic acids are a group of toxic phytochemicals found in the *Aristolochiaceae* plant family. These herbs used in Chinese medicine are associated with urothelial carcinoma and can cause end-stage renal failure. Aristolochic acid I, a potent nephrotoxin, is metabolized by several enzymes, including SULTs in the liver. This hepatic bioactivation has been shown *in vitro* in a microphysiological system to be the key factor in aristolochic acid I toxicity through the formation of the sulfate conjugate sulfonyloxyaristolactam (Chang et al., 2017). Even more importantly, transporters play a critical role in the renal toxicity of this compound. Sulfonyloxyaristolactam is transported out of hepatocytes by MRP3 and MRP4 and concentrated by OAT1 and OAT3 from blood, and by OAT4 from urine, into the proximal tubular epithelial cells, where this toxin reacts to form DNA adducts at high levels. Inhibition of OATs by probenecid, decreased the toxicity in kidney cells by 50–60% in the microphysiological *in vitro* system (Chang et al., 2017).

Transporter-mediated enhancement of toxicity has also been observed with other reactive sulfate conjugates. 1-sulfooxymethylpyrene is a sulfate metabolite of 1-methylpyrene, a procarcinogen present for example in cigarette smoke. *In vitro*, 1-sulfooxymethylpyrene accumulated into OAT1-or OAT3-overexpressing cells resulting in 4.6- and 3.0-fold higher DNA adduct formation, respectively, compared to control cells (Bakhiya et al., 2006). OAT-inhibitor probenecid abolished this effect, indicating that the OAT-mediated uptake of 1-sulfooxymethylpyrene is important for its renal toxicity. Similar results have been seen with 5-sulfooxymethylfurfural, a reactive sulfate metabolite of inactive 5-hydroxymethylfurfural, which is found in many foods (Bakhiya et al., 2009). Like 1-sulfooxymethylpyrene, 5-sulfooxymethylfurfural is a substrate for OAT1 and OAT3. OAT-mediated uptake was found to significantly increase its cytotoxicity, whereas the inhibition of OATs by probenecid reduced the cytotoxic effects (Bakhiya et al., 2009).

## 8 Effects of Pharmacogenetics on Conjugate Disposition

Genetic variation in transporter genes can cause alterations in transporter abundance, localization or function, leading to altered disposition of their substrates, including glucuronide and sulfate conjugates. Transporter variants have a well-described role in two benign bilirubin syndromes involving bilirubin conjugates: 1) In people with Rotor syndrome, decreased hepatic uptake by OATP1B1 and OATP1B3 loss-of function variants increases circulating levels of total bilirubin (van de Steeg et al., 2012) and 2) loss-of-function variants of MRP2 cause the Dubin-Johnson syndrome, where decreased biliary excretion increases conjugated bilirubin levels in the serum and liver (Kartenbeck et al., 1996). Transporter genotype also influences endogenous sulfate conjugates, as healthy volunteers with OATP1B1 *15/*15 haplotype (c.388A>G, rs2306283; and c.521T>C, rs4149056) had >1.7-fold higher AUCs of GCDCA-S, lithocholate-S, glycolithocholate-S and taurolithocholate-S compared to OATP1B1 *1b/*1b (c.388A>G) (Mori et al., 2019). Furthermore, in a recent genome-wide association study on metabolomic data, genetic variants of an orphan transporter, SLC22A24, were found to be associated with androsterone-G and etiocholanolone-G levels (Yee et al., 2019). In addition to affecting endogenous compounds, there is increasing evidence showing that transporter pharmacogenetics contribute to interindividual variability in drug pharmacokinetics (Giacomini et al., 2013). Although less is known about the effect of genetic variation on the disposition of phase II conjugates than on parent drugs, several cases where transporter variants cause altered drug glucuronide or sulfate disposition have been found.

### 8.1 Ezetimibe-G

Ezetimibe, a cholesterol-lowering drug acting primarily in the intestine, is extensively glucuronidated in enterocytes to form ezetimibe-G. Ezetimibe-G is a substrate of several transporters including OATP1B1, OATP2B1, and MRP2 (Zamek-Gliszczynski et al., 2014) (Table 1). Since ezetimibe-G is formed in the intestine, hepatic uptake could be a rate-limiting step for enterohepatic recycling as only the glucuronide is a substrate of OATP1B1 (Oswald et al., 2008). The cellular uptake of ezetimibe-G was reduced in cells expressing the OATP1B1 *1b and *5 (c.521T>C, rs4149056) haplotypes compared to OATP1B1 *1a (reference genotype). Healthy volunteers carrying OATP1B1 *1b/*1b had a 50% lower AUC of ezetimibe compared to carriers of *1a/*1a, and a trend for a higher ezetimibe-G AUC as well as increased amount of ezetimibe-G excreted in urine. In addition, *5 and *15 carriers had decreased excretion of ezetimibe into feces. These results suggest that decreased OATP1B1 function shifts ezetimibe excretion from biliary to renal and reduces enterohepatic recycling. However, these changes did not significantly affect the pharmacodynamic effect of ezetimibe in a study with healthy volunteers (Oswald et al., 2008).

### 8.2 Morphine-Gs

The opioid analgesic morphine is primarily eliminated by glucuronidation in the liver and the subsequent glucuronides, morphine-3-G and morphine-6-G, are substrates of MRP2 and MRP3, which transport the glucuronides to the bile and the systemic circulation, respectively (Zelcer et al., 2005). The disposition of morphine-6-G is of particular interest since it is an active metabolite. The appearance of morphine glucuronides in the systemic circulation appears to be associated with a promoter region variant of *ABCC3* (c.-211C>T, rs4793665) in pediatric patients, with the CC genotypes having a higher glucuronide level than the CT and TT genotypes (Venkatasubramanian et al., 2014; Chidambaran et al., 2017). Even though morphine is not known to be a substrate of MRP3, a link between apparent decreased morphine clearance and the c.-211C>T variant has been found in pediatric patients (Hahn et al., 2020). This is suggested to be due to a shift from the urinary excretion pathway to biliary excretion: Patients with the CT or TT genotypes had lower MRP3 activity than those with the CC genotype, leading to increased excretion of the glucuronides to the bile by MRP2, and a subsequent increase in enterohepatic recycling and morphine exposure. Furthermore, some *ABCC3* intronic variants were found to be associated with a longer postoperative unit care stay, due to respiratory depression as a side effect of morphine treatment (Chidambaran et al., 2017).

### 8.3 Raloxifene-Gs

As described in Section 4.2, raloxifene undergoes high intestinal and hepatic metabolism to several glucuronide conjugates. Although direct *in vitro* evidence is still missing, it is expected that OATP1B1 and OATP1B3 are required for the uptake of raloxifene-Gs into hepatocytes and subsequent biliary excretion by apical efflux transporters (Trdan Lušin et al., 2012a, 2012b; Kosaka et al., 2015). The role of OATP1B1 is supported by the association of OATP1B1 *1b haplotype with higher serum concentrations of raloxifene species (raloxifene, raloxifene-6,4′-di-G and total raloxifene) compared with OATP1B1 *1a in a study with 57 postmenopausal women treated with raloxifene for 12 months (Trdan Lušin et al., 2012b). Decreased hepatic uptake of raloxifene-Gs by the low-function *1b haplotype would explain the observed increase in the systemic concentrations of the raloxifene species. This haplotype was also linked to a higher decrease in serum C-terminal telopeptide fragments of type I collagen, a bone resorption marker, indicating a better therapeutic effect, probably due to the increased total systemic raloxifene exposure (Trdan Lušin et al., 2012b). The *SLCO1B1* c.521T>C polymorphism, which is included in the *5 and *15 haplotypes, did not significantly influence the pharmacokinetics or pharmacodynamics of raloxifene species in this study. No significant association was found either for *SLCO1B3* int7C>G (rs17680137) or for efflux transporter polymorphisms *ABCB1* c.3435C>T (rs1045642) or *ABCC2* c.3972C>T (rs3740066) (Trdan Lušin et al., 2012a; 2012b).

### 8.4 MPA-G

Mycophenolate mofetil is an immunosuppressive pro-drug that is commonly used in solid organ transplantations. The active form, mycophenolic acid, undergoes extensive glucuronidation in the liver, and the main metabolite, MPA-G, is pharmacologically inactive (Lamba et al., 2014). Additionally, a pharmacologically active acyl glucuronide is formed as a minor metabolite. Active secretion in the kidney is the main elimination pathway of MPA-G, but a significant portion of MPA-G is excreted to the bile and undergoes enterohepatic recycling, so that only a small portion of MPA-G is found in feces (Bullingham et al., 1998). Systemic MPA-G is taken up by OATP1B1 and OATP1B3 in hepatocytes, while MRP2 mediates MPA-G excretion into bile and is involved in secreting MPA-G into urine (Naesens et al., 2006; Picard et al., 2010; Matsunaga et al., 2014). OATP1B1 and OATP1B3 genotypes were predictive for MPA-G exposure in a study with 80 Japanese renal transplant patients (Miura et al., 2008). Furthermore, the decreased function *ABCG2* polymorphism c.421C>A (rs2231142) caused ≈30% increase in median MPA-G AUC, but MPA-G transport by BCRP remains to be verified *in vitro*. The OATP1B3 haplotype c.334T>G–c.699G>A (rs4149117 and rs7311358, respectively) resulted in decreased MPA AUC and increased MPA-G/MPA AUC ratio in renal transplant patients, while studied OATP1B1 polymorphisms had no effect on MPA or MPA-G (Picard et al., 2010). The decrease in MPA AUC is suggested to result from reduced hepatic uptake of MPA-G and subsequent enterohepatic recycling, leading to lower AUC and less frequent adverse reactions. *In vitro* and *in vivo* studies support this hypothesis, as uptake of MPA-G was reduced in OATP1B3 c.334T>G–c.699G>A expressing cells (Picard et al., 2010) and this haplotype was associated with lower survival and increased risk for non-minimal acute rejection (Tague et al., 2020). Although *in vivo* pharmacokinetic evidence is missing, MPA-G uptake is also reduced in OATP1B1 *5 expressing cells *in vitro* and the OATP1B1 *5 haplotype protected patients from MPA-related adverse reactions after renal transplantation (Michelon et al., 2010). The effect of MRP2 polymorphisms on MPA-G exposure is more contradictory (Lamba et al., 2014).

## 9 Effects of Disease on Conjugate Disposition

Diseases affecting the intestine, liver or kidney can alter the absorption and excretion of drugs and their metabolites, either through physiological changes (e.g. in blood flow, protein binding), or through altered function of metabolic enzymes and transporters. The pharmacokinetic impact of disease-mediated changes in drug transporters has recently been reviewed by Evers et al. (2018). However, clinical evidence for transporter-mediated alterations in drug conjugate exposure in disease is still scarce even though conjugates may be sensitive to changes in transport due to their low passive permeability.

### 9.1 Liver Disease

Liver disease is known to alter the metabolic capacity of hepatocytes and hepatic clearance may be impacted by changes in liver blood flow rates or even intra-hepatic shunting of blood in severe cases of liver impairment (Drozdzik et al., 2021). In addition, there is increasing evidence for changes in transporter protein levels in different types of liver disease (Drozdzik et al., 2020). For example, levels of several hepatic uptake transporters, as well as MRP2, tend to decrease with increasing liver disease severity, whereas OCT1, MRP3, MRP4 and P-gp levels tend to increase. Some changes, however, appear to be disease specific. The clinical significance of these changes in transporter levels is poorly characterized and many studies have relied on data from animal models of liver disease (Thakkar et al., 2017). Animal models have been useful, for example, to elucidate the role of MRP2 in estradiol-17-G induced cholestasis (Huang et al., 2000).

Two examples of altered conjugate disposition in hepatic disease come from studies of nononalcoholic steatohepatitis (NASH), the advanced stage of nonalcoholic liver disease. In patients with NASH, systemic exposure to morphine-3-G, morphine-6-G and acetaminophen-G is increased (Canet et al., 2015; Ferslew et al., 2015). This is likely due to the increased abundance of hepatic MRP3 and mislocalization of MRP2 in NASH, which results in increased efflux into the blood circulation and reduced biliary excretion (Hardwick et al., 2011; Vildhede et al., 2020; Sjöstedt et al., 2021). On the other hand, an observed trend for decreasing acetaminophen sulfate levels could be due to altered sulfonation activity caused by impaired sulfur activation in NASH (Canet et al., 2015), which highlights the importance (and challenge) of distinguishing changes in metabolite formation from transporter-mediated alterations. More studies are needed to provide further evidence on how conjugate disposition is impacted by liver disease and to elucidate, for example, whether the observed shift from biliary to sinusoidal clearance in NASH and other diseases has a significant effect on enterohepatic recycling and drug exposure.

### 9.2 Renal Disease

There is little information on transporter levels in renal disease in humans, although a decrease in OAT1 expression at the mRNA level has been reported (Sakurai et al., 2004). Renal disease and decreased kidney function are known to increase the exposure to several drug glucuronides (Verbeeck, 1982). For example, oxazepam and oxazepam-G are typically found at comparable levels in healthy subjects, but the oxazepam-G to oxazepam ratio was shown to increase to up to 50 due to an increase in oxazepam-G in patients with renal insufficiency (Odar-Cederlöf et al., 1977). Further, in a study with renal failure patients, the concentrations of propranolol-Gs were up to 18-fold higher compared with patients with normal renal function (Stone and Walle, 1980). These two examples clearly highlight the possibility of high accumulation of drug conjugates in renal diseases. Moreover, fecal excretion and enterohepatic recycling may become more pronounced for conjugates primarily eliminated via the kidney in healthy individuals if excretion is shifted to the biliary route, as shown for example for oxazepam (Odar-Cederlöf et al., 1977).

An age-related decrease in active renal secretion was suggested to result in a 1.9- to 2.5-fold reduction in the renal clearance of propafenone-Gs in older compared to younger subjects with normal renal function (Fromm et al., 1995), but the transporters involved in secretion have not been identified. Zhong et al. (2014) showed that severe renal impairment increased exposure to sulfate and glucuronide conjugates of morinidazole ≥15-fold and identified them as OAT1 and OAT3 substrates. Therefore, decreased transporter function could explain the reduction of their renal clearance. The challenge in renal disease is to distinguish whether pharmacokinetic changes are caused solely by changes in glomerular filtration rate or by alterations in transporter-mediated renal excretion. The distinction is further complicated by the use of serum creatinine to estimate glomerular filtration rate. Transporters are involved in creatinine clearance and changes in serum creatinine may in some cases be attributed to altered transport activity (Chu et al., 2016). Furthermore, transporter function may be altered in kidney disease both due to altered expression as well as inhibition by the uremic toxins that accumulate with declined kidney function (Table 3).

**TABLE 3:** Interaction of drug transporters with uremic toxins in transporter overexpression systems.

Uremic toxin^a^	Influx transporters^b^	Efflux transporters^b^	Reference
Indoxyl-G	Inhibitor		Hsueh et al. (2016); Cheung et al. (2017)
	OAT1 (32% at 950 µM)		
	OAT3 (IC_50_ = 670 µM)		
	OCT2 (IC_50_ = 58 µM)		
Indoxyl-S	Inhibitor	Inhibitor	Motojima et al. (2002); Enomoto et al. (2003); Deguchi et al. (2004); Mutsaers et al. (2011); Reyes and Benet (2011); Sato T. et al. (2014); Hsueh et al. (2016); Katsube et al. (2017); Kong et al. (2017); Lin et al. (2018); Morimoto et al. (2018); Takada et al. (2018); Weigand et al. (2019)
	NTCP (26% at 500 µM)	BCRP (K_i_ = 500 µM)	
	OAT1 (Ki = 13–23 µM)	MRP2 (40% at 3 mM)	
	OAT2 (20% at 1000 µM)	MRP4 (K_i_ = 1000 µM)	
	OAT3 (K_i_ = 169–183 µM)	BSEP (-)	
	OAT4 (K_i_ = 181 µM)	MRP3 (-)	
	OATP1B1 (IC_50_ = 1061–2700 µM)	P-gp (-)	
	OATP1B3 (IC_50_ = 1300 µM)		
	OATP2B1 (30% at 400 µM)		
	OCT1 (-)		
	OCT2 (-)		
	Substrate	Substrate	
	OAT1	BCRP	
	OAT3	MRP2 (-)	
	OAT4	P-gp (-)	
	OATP1B1 (-)		
	OATP1B3 (-)		
p-Cresyl-G	Inhibitor	Inhibitor	Mutsaers et al. (2015); Weigand et al. (2019)
	OATP1B1 (24% at 500 µM)	MRP4 (73% at 1 mM)	
	OATP1B3 (18% at 500 µM)	BCRP (-)	
	NTCP (-)	BSEP (-)	
		Substrate	
		BCRP	
		MRP4 (-)	
p-Cresyl-S	Inhibitor	Inhibitor	Watanabe et al. (2014); Mutsaers et al. (2015); Hsueh et al. (2016); Weigand et al. (2019)
	NTCP (54% at 500 µM)	BCRP (24% at 1 mM)	
	OAT1 (IC_50_ = 210–690 µM)	MRP4 (40% at 1 mM)	
	OAT3 (IC_50_ = 200–485 µM)	BSEP (-)	
	OATP1B3 (16% at 500 µM)OATP1B1 (-)	MRP3 (-)	
	
	Substrate	Substrate	
	OAT1	BCRP	
	OAT3	MRP4 (-)	

a Reported total mean plasma levels of indoxyl-G, indoxyl-S, p-cresyl-G, and p-cresyl-S in end stage renal disease patients are 9, 110, 44, and 675 µM, respectively Duranton et al. (2012).

b Inhibition reported as inhibitory constant (K_i_), half-maximal inhibitory concentration (IC_50_) or inhibition percentage at a defined concentration of the inhibitor. (-) denotes results showing no interaction.

Uremic toxins include several glucuronide and sulfate conjugates. The most noteworthy of these are indoxyl-S and p-cresol-S because of their role in the progression of chronic kidney disease, cardiovascular disease and interactions with transport proteins (Sirich et al., 2014). Both also exist as glucuronide conjugates. Indoxyl-S and p-cresol-S are eliminated via renal excretion and impaired kidney function can elevate their plasma levels by more than 40- and 10-fold, respectively (Duranton et al., 2012). Renal excretion of uremic toxins is facilitated by OAT1, OAT3, OAT4 and BCRP and they can inhibit multiple transporters *in vitro* (Table 3). The ratio of plasma concentrations to IC_50_ values of indoxyl-S and p-cresyl-S suggest potential for clinically significant inhibition. For example, inhibition by uremic toxins was proposed to be the mechanism for reduced clearance of morinidazole conjugates in a rat model of chronic renal failure (Kong et al., 2017). Notably, the effects of uremic toxins are not limited to renal transporters, but may also alter hepatic clearance by inhibiting OATP-mediated uptake into the liver (Tan et al., 2018), as indoxyl-S and p-cresyl-S have been shown *in vitro* to inhibit OATPs (Table 3). Furthermore, the accumulation of uremic toxins can possibly lead to displacement of other drugs from albumin and this combined with hypoalbuminemia common in chronic kidney disease patients may increase the unbound fraction of drugs.

### 9.3 Intestinal Disease

Drug absorption is affected greatly by intestinal physiology, which can be altered in disease (Stillhart et al., 2020). Transporters in the intestine may have a smaller role than in the liver and kidney on conjugate disposition except if the conjugation occurs in the enterocytes. In this case, disease-associated changes in transporter levels or function may alter the fraction of conjugate reaching the systemic circulation, but limited information on transporter changes is available. For example, inflammation in ulcerative colitis has been shown to decrease mRNA, but not protein, levels of P-gp and BCRP and increase *SLCO2B1* mRNA levels when compared with non-inflamed tissue (Erdmann et al., 2019). Jahnel et al. (2014) reported decreased *ABCG2* mRNA levels in Crohn’s disease compared to healthy controls, whereas both *ABCC3* and *ABCC4* mRNA was decreased in ulcerative colitis in their study. However, the *in vivo* effect of these transporter changes on conjugate kinetics remains to be elucidated. Besides transporter changes, any diseases affecting the gut microbiome and bacterial β-glucuronidase activity could affect conjugate and parent drug disposition by decreasing intestinal deconjugation and leading to impaired enterohepatic recycling.

## 10 Conjugate Disposition Studies in Preclinical Animals

*In vivo* studies are needed to understand how the complex interplay of metabolism and transport affects the overall disposition and elimination patterns of drugs and their metabolites. However, plasma, urine and fecal samples from human subjects are often insufficient for resolving detailed mechanisms in disposition. Studies in preclinical animals allow more invasive sampling, such as bile collection, that is not typically feasible in human subjects. Important mechanistic information can also be obtained when performing studies in animals where specific transporters have been knocked out or by using humanized rodent models (Durmus et al., 2016). Examples of transporter-mediated conjugate disposition studies in efflux knockout rodents have been discussed previously by (Zamek-Gliszczynski et al., 2006) and examples for uptake transporters are summarized in Table 4.

**TABLE 4:** Studies in knockout (KO) mice supporting the significance of uptake transporters in the disposition of glucuronide and sulfate metabolites of drugs and other compounds.

Compound	KO model	Summary	Reference
Bilirubin-Gs (endogenous)	Oatp1a/b	High plasma concentration of bilirubin-Gs in KO mice, while they were absent in wild type (WT) mice. Biliary output of the glucuronides was reduced 2-fold in KO mice. No excretion of glucuronides in urine in the WT mice, while high excretion in the KO mice.	van de Steeg et al. (2010)
Bilirubin-Gs (endogenous)	Oatp1a/b with and without Mrp2, Mrp3 and Mrp2/Mrp3	Oatp1a/b KO increased the plasma level of bilirubin-mono-G ∼50-fold. Of the other double KOs, only Oatp/Mrp2 KO further increased the plasma concentration (up to 150-fold) in comparison to WT mice. Mrp2 KO by itself resulted only in a 4-fold increase in comparison to WT mice. Similarly, urinary excretion increased for the glucuronides in all KO mice strains, but the difference between Mrp2 and Oatp/Mrp2 KO strains was almost 100-fold. Oatp1a/b KO reduced the biliary excretion of the glucuronides only 2-fold.	van de Steeg et al. (2012)
Estradiol-17-G (intravenous)	Oatp1a1	Oatp1a1 KO resulted in 1.5-fold increase in the initial AUC of estradiol-17-G between 1 and 2 min. Oatp1a4 KO did not have an effect.	Gong et al. (2011)
	Oatp1a4		
Metabolomics (endogenous)	Oat1	9-fold and 3-fold higher plasma concentrations of indoxyl-S and phenyl-S in the KO mice in comparison to WT mice. 10-fold lower amino-cresol-S in KO mouse urine.	Wikoff et al. (2011)
Regorafenib-G (oral regorafenib)	Oatp1b2	6-fold higher regorafenib-G AUC in the KO mice. No change in the exposure of regorafenib, regorafenib-N-oxide nor N-desmethyl-regorafenib-N-oxide.	Fu et al. (2018)
Sorafenib-G (oral sorafenib	Oatp1b2	7–23-fold higher AUC of sorafenib-G in KO mice compared to WT mice. AUC of sorafenib or sorafenib-N-oxide was unchanged.	Fu et al. (2019)
Regorafenib-G (oral regorafenib)		4–9-fold higher AUC of regorafenib-G in the KO mice compared to WT mice. Regorafenib and regorafenib-N-oxide levels were unchanged.	
Sorafenib-G (oral sorafenib)	Oat1b2 and Oatp1a/1b	5-fold higher AUC of sorafenib-G in Oat1b2 KO mice, while no change in the AUC of sorafenib-N-oxide and only slight change in the AUC of sorafenib. Liver/plasma ratio of sorafenib-G was reduced 6-fold in Oatp1b2 KO mice. 29-fold higher AUC in Oatp1a/1b KO mice, while no change in the AUC of sorafenib-N-oxide and only slight change in the AUC of sorafenib.	Zimmerman et al. (2013)
Sorafenib-G (oral sorafenib)	Oatp1a/b with and without Mrp2, Mrp3 and Mrp2/Mrp3	KO of Oatp1a/b, Oatp1a/b with Mrp2, Oatp1a/b with Mrp3, and Oatp1a/b with Mrp2 and Mrp3 resulted in 72-, 906-, 38- and 644-fold increase in the AUC of sorafenib-G in comparison to WT mice. Oatp1a/b KO increased the liver concentration of sorafenib-G only 1.5-fold.	Vasilyeva et al. (2015)
Sorafenib-G (oral sorafenib)	Oat1b2	8-fold higher AUC of sorafenib-G in the KO mice. The plasma AUC of sorafenib and sorafenib-N-oxide were unchanged. 10-fold lower liver/plasma ratio of sorafenib-G in the KO mice.	Bins et al. (2017)

Knockout animal studies have been instrumental in clarifying the roles and interplay of uptake and efflux transporters in the liver. Studies in Oatp1a/b knockout mice revealed that the glucuronides of bilirubin undergo Oatp1a/b-mediated shuttling between hepatocytes, which was termed hepatocyte hopping (van de Steeg et al., 2010). Later, sorafenib-G was also found to exhibit similar behavior (Vasilyeva et al., 2015). In mice, Mrp2, Oatp, Oatp;Mrp2 and Oatp;Mrp2;Mrp3 knockouts all led to a substantial (38–906-fold) increase in sorafenib-G plasma concentrations, while the increase in liver concentrations was more modest (1.5 – 3-fold). Furthermore, knockout mice were used to resolve the roles of sinusoidal and canalicular transporters in diclofenac-AG disposition (Lagas et al., 2010).

The above examples highlight the utility of preclinical *in vivo* studies, especially in knockout animals. However, compensatory changes in knockout animal models should be considered as it is well known, for example, that decreased MRP2/Mrp2 function in humans and rodents leads to an increase in hepatic MRP3/Mrp3 protein levels (König et al., 1999; Kuroda et al., 2004). Inherent interspecies differences may also complicate the interpretation and extrapolation of preclinical data on drug conjugates to humans (Zamek-Gliszczynski et al., 2006). For example, rats do not have a gall bladder and human OATP isoforms do not have direct orthologs in the rat. Based on its specific expression in the liver, rat Oatp1b2 is considered to correspond to OATP1B1 and OATP1B3, even though there are differences in their substrate specificity (Cattori et al., 2001). Differences also exist in transporter abundance. According to a proteomics study across liver samples from human, monkey, dog and rat, OATPs/Oatps were the most abundant transporters, but their contribution to the total abundance varied from 29% in humans to 69% in dogs, with the total Oatp expression being approximately 4-fold higher in rat than in human (Wang L. et al., 2015). In rat livers, Mrp2 protein levels were over 5-fold compared to human livers, whereas Bcrp and Mrp3 abundances were below the detection limit in rat livers, unlike in humans. High expression of Oatps and Mrp2 in rats may contribute to the pronounced biliary excretion observed in rats compared with humans (Grime and Paine, 2013). For the kidney, transporters tended to have higher abundance in kidney cortices in monkey, dog, rat and mice than in humans (Basit et al., 2019). Importantly, regarding localization differences, Oat2 is found only on the apical membrane of renal proximal tubule cells in rats, while human OAT2 is found primarily on the basolateral membrane (Shen et al., 2015).

In addition to differences in transporter levels, substrate specificity can vary between species. Data for species differences in transport of sulfate and glucuronide conjugates is sparse. In our literature search, only a handful of drug conjugates had data available for humans and preclinical species, but the data suggests that drug conjugates that are substrates of human transporters are generally also substrates of the ortholog proteins (Supplementary Table S1). An exception to this is, for example, raloxifene-6-G, which is a substrate of rat Mrp2 and its disposition is altered in Mrp2-deficient EHBR rats (Kosaka et al., 2015). However, it does not appear to be a substrate of human MRP2, as determined by an indirect *in vitro* assay measuring ATP hydrolysis in the presence of raloxifene-6-G (Trdan Lušin et al., 2012a). Similarly, paroxetine M1-G is a substrate of rat MRP2, but not human MRP2 (Matsunaga et al., 2013). Species differences in transporter affinity and inhibition potency may be significant when evaluating drug safety. For example, the lower inhibition potency of fasiglifam-AG towards transporters in rats than in humans may have contributed to the late identification of fasiglifam DILI risk (Otieno et al., 2018).

Differences in UGTs and SULTs also need to be considered when studying drugs that undergo conjugation, since alterations in both transport and metabolism can affect the observed exposure of conjugated metabolites. In the case of fasiglifam, lower formation of fasiglifam-AG in rats may have further contributed to differences in observed DILI risk (Otieno et al., 2018). Species differences in metabolism can also change the overall disposition patterns. Maribavir, a novel agent for the management of human cytomegalovirus infection, is primarily metabolized via glucuronidation in non-human primates and undergoes biliary excretion and enterohepatic recycling, whereas only 20% of the dose is glucuronidated in human hepatocytes and no direct glucuronides were found in human feces (Sun and Welty, 2021).

Species differences in UGTs have been characterized (Oda et al., 2015; Fujiwara et al., 2018). The *UGT1A* gene family is mostly conserved among species and the isoforms are generally expressed in the same tissues in humans and rodents, whereas assigning orthologs of human *UGT2* genes in other species is more difficult. Even for UGT1As, it should be noted that Ugt1a8 and Ugt1a10 are not intestine-specific isoforms in mice as they are in humans (Fujiwara et al., 2018). More importantly, N-glucuronidation of tertiary amines is very low in mice and rats (Kaivosaari et al., 2011). Most substrates that are metabolized by human UGT1 isoforms in humans are also glucuronidated in rodents, but the clearance may vary several fold. For instance, the intrinsic formation clearance of furosemide-AG and naproxen-AG are approximately 5-fold and 3-fold, respectively, in mice compared to humans (Kutsuno et al., 2013). Conversely, MPA glucuronidation occurs at a 5-fold rate in humans compared to rat (Shiratani et al., 2008). Differences also exist for UGT2B7, which catalyzes morphine glucuronidation in humans. In rats and mice, morphine-3-G is formed at approximately 17- and 29-fold higher rates, respectively, than in humans, whereas morphine-6-G, which is formed in humans, could not be detected in rats and mice (Soars et al., 2001; Shiratani et al., 2008). Less data is available comparing SULT expression and activity in preclinical species, but some evidence of differences exists. For example, the *O*-demethyl phase I metabolite of apixaban is sulfated in human S9 liver fractions with over 50-fold higher rate than in rat and over 600-fold higher than in mouse S9 fractions, whereas the sulfate formation rate was more similar to humans in dogs and monkeys (Wang et al., 2009).

## 11 Discussion

Phase II glucuronide and sulfate metabolites are typically less active than their parent drugs, but these metabolites can still contribute significantly to drug exposure, for instance through enterohepatic recycling or DDIs, and may even cause toxicity. Since transporters are vital determinants of the disposition of poorly permeable phase II conjugates, it is important to understand the interactions between transporters and these conjugates. By characterizing the role of different transporters and the sum of uptake and efflux transport across membranes, the fate of a compound in the body can be explained (see Section 4). Despite increased study within the last decade, there are still many unanswered questions regarding transport of sulfate and glucuronide conjugates. Interactions with efflux transporters have been better characterized than with uptake transporters, but there are still many conjugates with a paucity of information regarding both uptake and efflux. The large number of endogenous and natural compound conjugates also identified in our literature search further highlight the prevalence of interactions between phase II conjugates and drug transporters (Supplementary Table S1).

Among efflux transporters, BCRP, MRP2 and MRP3 appear to have the most conjugate substrates, while P-gp, which transports many parent drugs, does not seem to influence the disposition of conjugates (Table 1). The relative transport activity of MRP2 on the apical and MRP3 and OATP1Bs on the basolateral membranes of hepatocytes appears particularly important for determining the excretion routes for conjugated drugs. Even for glucuronide conjugates undergoing biliary excretion, hepatic uptake may be the rate-limiting factor, especially for glucuronides formed in the intestine, such as ezetimibe and raloxifene glucuronides (Oswald et al., 2008; Trdan Lušin et al., 2012b). Due to the complex interplay between metabolism and transport, alterations in transporter activity can shift the excretion pathway and exposure of the parent drug and the metabolite, thereby affecting the safety and efficacy of the drug.

The role of phase II metabolites in DDIs is poorly characterized, partly since it can be difficult to distinguish the role of inhibition by the conjugate versus the parent drug in the clinical setting. Evaluating the likelihood of inhibition is especially challenging for efflux transporters as inhibition is likely related to intracellular concentrations, whereas uptake transporter inhibition is typically driven by extracellular (systemic) concentrations. Furthermore, both uptake and efflux transporters may play a role in determining intracellular concentrations of conjugates and may thus affect not only transporter inhibition, but also the likelihood of inhibition of metabolism. Conjugated drug metabolites may have been overlooked as possible perpetrators for DDIs (Table 2), particularly for renal uptake transporters, as a recent study identified several drug conjugate inhibitors of OAT1 and OAT3 (Zou et al., 2021). The clinical significance of this *in vitro* OAT inhibition by conjugates remains to be determined, but some conjugates have already been implicated in clinical DDIs (Zamek-Gliszczynski et al., 2014; Patel et al., 2016). Furthermore, endogenous glucuronide and sulfate bile acid conjugates have in recent years received interest as endogenous biomarkers for transporter inhibition in clinical studies for identifying DDI risk (Section 6.3).

Overall, there are still many challenges to solve when determining the mechanisms behind the disposition and effects of phase II conjugates in the body. As the potential impact of transporter interactions is recognized, the interactions of new drugs and their conjugates with transporters are extensively studied. However, the interactions and impact of conjugates of older drugs, natural products and many other compounds with transporters are unknown. *In vitro* studies can be used to identify transporter substrates and inhibitors, but other methods are needed in order to evaluate the *in vivo* impact. Due to the large substrate overlap between transporters and metabolic enzymes there is a lack of specific inhibitors, biomarkers and probe substrates to use for mechanistic *in vivo* studies. Although knockout animal models are useful to elucidate transporter impact, they have significant limitations due to species differences and compensatory changes in transporter levels. Pharmacogenetic studies can also help to gain mechanistic information on conjugate disposition, but should ideally include genotyping of all relevant transporters instead of focusing on a single pathway. Finally, physiologically-based pharmacokinetic modeling has the potential to integrate metabolism and transport data to elucidate their roles in different tissues and predict conjugate disposition and tissue concentrations even in complex cases, including e.g. those involving enterohepatic recycling.

In conclusion, the number of identified interactions between uptake and efflux transporters and phase II conjugates continues to increase. This increased understanding of transporter involvement will further improve our ability of predict drug disposition and possible interindividual variability due to intrinsic (e.g. genotype, disease) and extrinsic factors (e.g. concomitant medication).
